# Design and implementation of a high misalignment-tolerance wireless charger for an electric vehicle with control of the constant current/voltage charging

**DOI:** 10.1038/s41598-024-63952-6

**Published:** 2024-06-07

**Authors:** Venkatesan Ramakrishnan, Dominic Savio A, Balaji C, Narayanamoorthi R, Pradeep Vishnuram, Tiansheng Yang, Mohit Bajaj, Rajkumar Singh Rathore, Ievgen Zaitsev

**Affiliations:** 1 Department of Electrical and Electronics Engineering, SRM Institute of Science and Technology, Kattankulathur, Chennai, 603203 India; 2 University of South Wales, Llantwit Rd, Pontypridd, CF37 1DL UK; 3 Department of Electrical Engineering, Graphic Era (Deemed to Be University), Dehradun, 248002 India; 4 Hourani Center for Applied Scientific Research, Al-Ahliyya Amman University, Amman, Jordan; 5 Graphic Era Hill University, Dehradun, 248002 India; 6 Cardiff School of Technologies, Cardiff Metropolitan University, Cardiff, CF5 2YB UK; 7 Department of Theoretical Electrical Engineering and Diagnostics of Electrical Equipment, Institute of Electrodynamics, National Academy of Sciences of Ukraine, Peremogy, 56, Kyiv-57, 03680 Ukraine; 8 Center for Information-Analytical and Technical Support of Nuclear Power Facilities Monitoring of the National Academy of Sciences of Ukraine, Akademika Palladina Avenue, 34-A, Kyiv, Ukraine

**Keywords:** Wireless power transfer (WPT), Electric vehicle (EV), Coil tracking system, Misalignment tolerant, Constant current (CC)/Voltage (CV) charging, Energy science and technology, Engineering, Mathematics and computing

## Abstract

Wireless charging of Electric Vehicles (EVs) has been extensively researched in the realm of electric cars, offering a convenient method. Nonetheless, there has been a scarcity of experiments conducted on low-power electric vehicles. To establish a wireless power transfer system for an electric vehicle, optimal power and transmission efficiency necessitate arranging the coils coaxially. In wireless charging systems, coils often experience angular and lateral misalignments. In this paper, a new alignment strategy is introduced to tackle the misalignment problem between the transmitter and receiver coils in the wireless charging of Electric Vehicles (EVs). The study involves the design and analysis of a coil, considering factors such as mutual inductance and efficiency. Wireless coils with angular misalignment are modelled in Ansys Maxwell simulation software. The proposed practical EV system aims to align the coils using angular motion, effectively reducing misalignment during the parking of two-wheelers. This is achieved by tilting the transmitter coil in the desired direction. Furthermore, micro sensing coils are employed to identify misalignment and facilitate automatic alignment. Additionally, adopting a power control technique becomes essential to achieve both constant current (CC) and constant voltage (CV) modes during battery charging. Integrating CC and CV modes is crucial for efficiently charging lithium-ion batteries, ensuring prolonged lifespan and optimal capacity utilization. The developed system can improve the efficiency of the wireless charging system to 90.3% with a 24 V, 16 Ah Lithium Ion Phosphate (LiFePO4) battery at a 160 mm distance between the coils.

## Introduction

Electric Vehicles (EVs) represent a sustainable transportation solution for addressing the greenhouse gas emissions produced by traditional fuel-based vehicles. Both electric vehicles and electric bicycles (e-bikes) are particularly regarded as highly convenient modes of transportation in Smart Cities. Their attractiveness stems from their cost-effectiveness, low maintenance requirements, suitability for crowded environments, and the absence of the necessity for licenses or road taxes. Following the advent of Electric Vehicles (EVs), researchers shifted their focus towards optimizing EV chargers. Additionally, e-bikes exhibit the lowest CO2 emissions per mile, ranging from 2.6 to 5.0 g depending on the energy source of charging stations. This is notably lower compared to electric cars at 75 g and scooters at 136 g^1^.

EVs can be charged in both ways wired and wirelessly, wired charging also known as conductive charging a method of charging an EV by physically connecting a charging cable to the vehicle's charging port^2^. Wireless charging is becoming increasingly popular as a way to charge EVs because of its convenience and Capability to decrease the intricacy of charging infrastructure. Wireless charging obviates the necessity for tangible cords, plugs, and sockets. Charging an electric vehicle becomes effortless by parking it on a wireless charging pad, which may be conveniently installed in a garage or parking lot^3^. Resonant Wireless Power Transfer (WPT) is based on a system consisting of two magnetically linked coils called the primary and secondary coils. When the primary coil is energized, it generates a dynamic magnetic field that is connected to the grid. The precise placement of the secondary coil is essential, necessitating its intersection with a certain segment of the magnetic field that is produced. The secondary coil, which is linked to the Electric Vehicle (EV) battery, enables the charging of this component. The efficiency of power transfer is significantly affected by the relative location or misalignment of the main and secondary coils^4^. Inductive WPT has been extensively tested in many prototypes and trials, resulting in the determination that this technology has reached a mature stage^5^. However, there have been only a limited number of studies carried out with alternate vehicle types such as e-bikes. Implementing this technology in e-bikes is not simple when relying exclusively on expertise obtained in the context of electric cars. Weight and physical stability are key aspects to consider when it comes to e-bikes^6^.

In order to tackle this problem, scientists have investigated many methods, such as optimizing coil structure and implementing compensating topological configurations, to improve the efficiency of power transmission. Nevertheless, intricate coil configurations might result in supplementary losses and an escalation in matching circuits, resulting in unwarranted intricacy and oscillations in power^7^. Magnetic isolation sheets are essential components in wireless charging technology as they amplify the induced magnetic field and provide protection against coil interference. The combination of magnetic isolation and wave-absorbing materials can improve the effectiveness of the coil and increase the distance over which transmission can occur^8^. To mitigate transmission efficiency losses caused by misalignment in WPT systems, magnet spacers can be utilized. Furthermore, the inclusion of magnetic cores can offset the reduction in efficiency. By establishing a system for charging that can accept misalignment, corporations might help to lower the cost and difficulty of installing additional charging infrastructure, which could make simpler for more companies and neighborhoods to adopt EV charging.

Wireless Power Transfer (WPT) systems are specifically designed to accommodate the unique characteristics of e-bike batteries. Presently, the most commonly utilized battery types in e-bikes encompass Li-Ion, Li-Fe, and lithium-ion polymer (Li-Po) batteries^9^. The process of charging these batteries consists of a two-phase sequence. At first, the battery is charged with a Constant Current (CC) until it reaches a predetermined rated voltage level. In the subsequent phase, the charging transitions to Constant Voltage (CV). Throughout this process, the equivalent resistance of the battery also undergoes variations^10^. Furthermore, it is essential to ensure the continuity of the Constant Current (CC) mode and the stability of the regulated voltage in the Constant Voltage (CV) mode, even in the presence of deviations from the standard operating conditions of the system^11^.

The study referenced in^12^ deploys the power transmitter with two coils, where one coil is exclusively connected during the Constant Voltage (CV) mode. It is evident that these experiments utilize intricate coil structures and have yet to explore the optimal positioning of these components for maximum convenience.

The Series-Series topology, as employed in^13^, represents the most straightforward configuration. However, alternative multi-resonant approaches exist. In^14^, the authors introduce an S-LCC topology for controlling Constant Current (CC) to Constant Voltage (CV) charging. On the primary side, there is a switch that enables or disables a single capacitor for each mode. A reactive network is formed on the secondary side, consisting of two capacitors. Conclusions drawn in^15^, and^16^ emphasize the cost-effectiveness of the Series-Series configuration. In^17^, the authors implement a Series-Series topology, incorporating two separate capacitors. One capacitor is designated for the Constant Current (CC) mode on the primary side, while the other is allocated for the Constant Voltage (CV) mode.

The development of efficient and reliable wireless power transfer (WPT) systems has long been a focus in the field of electric vehicle (EV) technology. One of the main challenges faced in such systems is the issue of angular misalignment between the transmitter and receiver coils. Angular misalignment can lead to suboptimal power transfer, resulting in decreased system efficiency and potential damage to the EV's battery. Existing WPT systems typically do not address this issue adequately, leading to a significant loss in power transfer efficiency. There is a critical need for a solution that can accurately track and adjust for angular misalignment, ensuring optimal power transfer and overall system performance.

The significant contribution of this article were summarized as:

(i) Implementation of CC and CV charging method to the developed 500W WPT charging system for the developed low cost electric scooter.
(ii) Performance analysis of WPT system with angular misalignment using Ansys-Maxwell Finite Element Analysis (FEA) tool.
(iii) Design and implementation of tracking system to overcome the angular misalignment and achieves high misalignment tolerance in WPT system for EV charging.

The article is organized as follows: The basic principles and operation of a WPT system and proposed CC-CV-based WPT are presented in Section II. The simulation analysis of WPT is discussed in Section III. Section IV the proposed system not only addresses the challenges posed by angular misalignment of WPT coils. Section V's emphasis on incorporating angular motion to align the coils demonstrates a strategic approach to mitigating misalignment issues during the parking of two-wheelers implemented using hardware setup.

## Principle of WPT system

**Figure 1:**
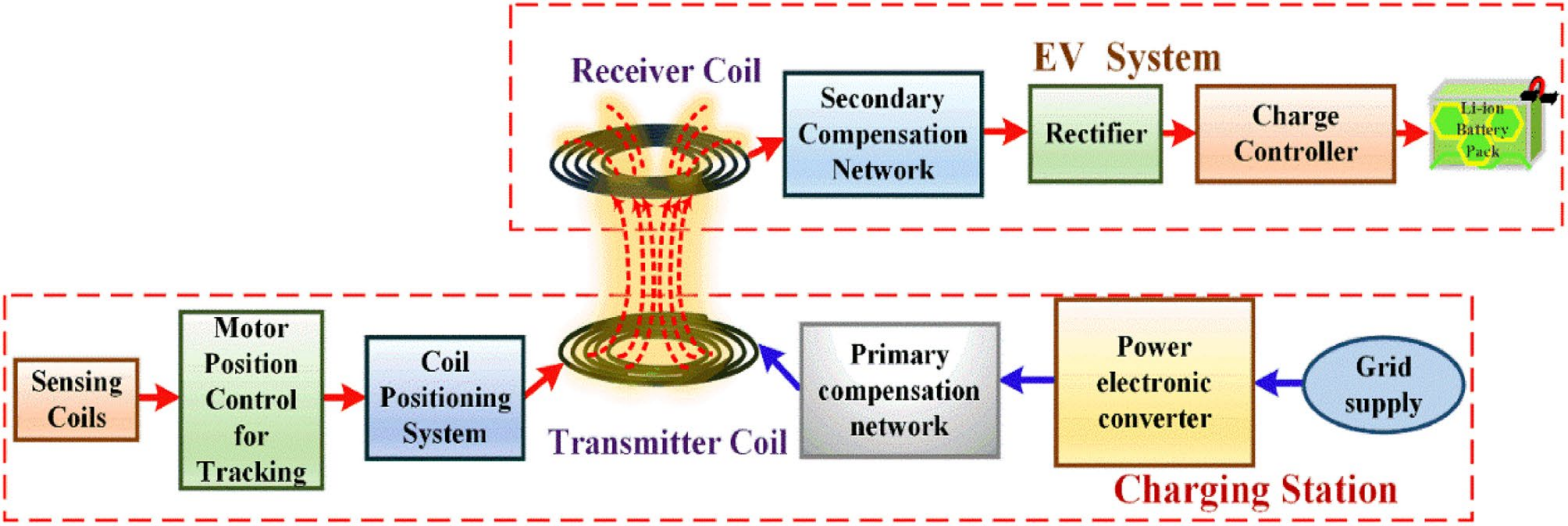
Block diagram for wireless charging of an EV with tracking mechanism.

Wireless power transfer operates based on electromagnetic induction. When an electric current passes through a conductor, it generates a magnetic field. This magnetic field induces an electromotive force (EMF) in nearby conductors when they interact. As a result, power can be transferred without the need for direct physical connections. This approach provides convenient means of charging EV, with rise of EV market. Penetration of EV increases the necessity of integrating Electric Vehicles (EVs) and renewable energy sources (RESs) within hybrid micro grids serves to prevent grid overloading, and efficient power management in the charging station facilitates effective charging^18^. Figure 1 shows the general block diagram of wireless power transfer with proposed tracking mechanism to charge an EV from the utility grid. The coupling between transmitter (Tx) and receiver (Rx) is improved by exciting the coil with high frequency AC source. Which is generated by power electronic converter powered by grid. The resonant condition is achieved and maintained by compensation circuits. This resonated high frequency AC is transferred to the receiver coil my mutual inductance. The received AC is rectified and regulated to charge the battery^19^.

Tracking mechanism is accomplished by using dual sensing coils, which aligns the transmitter pad with receiver pad through stepper motor and its driver controller. To achieve maximum transfer range, the coils need to be tightly coupled. The transmission efficiency of the coil is influenced by its internal resistance^20^. The coupling coefficient (k) is a measure of strength of the coupling between the coils, which is related to mutual inductance (M) as given by Eq. (1).

1 $$L = \frac{\phi (i)}{i},M = k\sqrt {L_{1} L_{2} }$$

To achieve maximum power transfer, the majority of the magnetic flux created by the transmitter should be linked to the receiver coil and the excess flux which is leaked to the environment must be well maintained and that should not affect the surrounding environment ^21^.

In wireless power transfer system the transmitter and receiver is the primary component in the process of power transfer. Here air is acting as a core for the transmission due to its high permeability the coupling between transmitter and receiver is weak (Loosely coupled). In such loosely coupled magnetic circuits power transmission is also poor. Which is improved by energizing the magnetic circuit with high frequency source and make resonance in the magnetic circuit. In case of circular pad type coupler flux density is maximum at the circular area, therefore proper alignment is necessary^22,23^. In systems utilizing inductively coupled power transfer, the magnetic field produced by transmitter coil, which evolves with the current flow, supports WPT. Together with components like rectifiers and compensation networks, this setup enables efficient energy transfer and successful better charging^24,25^.

### Analysis of two coil WPT system

**Figure 2:**
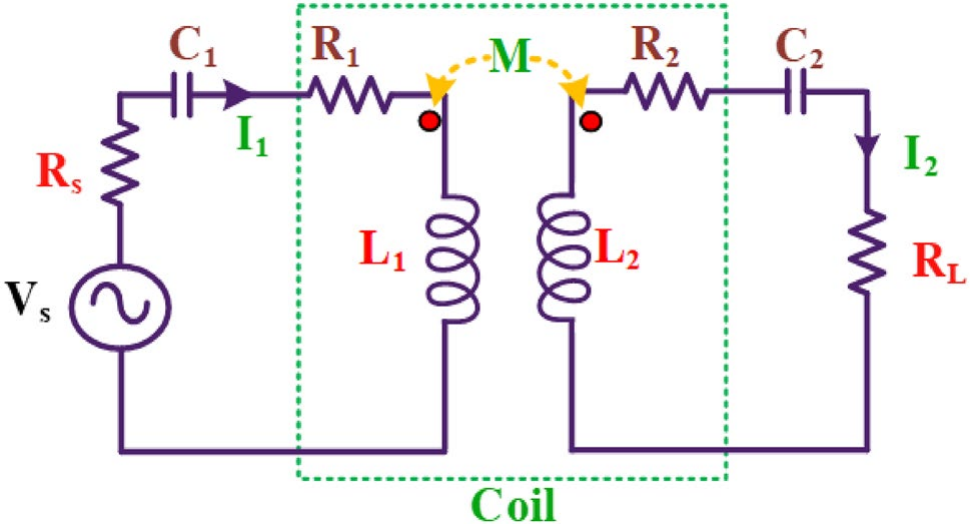
Equivalent circuit of WPT Series-Series compensation network.

The Inductive Power Transfer System is represented as an equivalent circuit, illustrated in Fig. 2. R_1_ and L_1_ denote the resistance and inductance of the transmitter coil, while C_1_ serves as the capacitance for achieving resonance in the circuit. On the receiver coil side, R_2_ and L_2_ represent the resistance and inductance, with C_2_ acting as the resonating capacitance. The efficiency of power transfer is contingent on the coupling factor between the transmitter and receiver coils.

In this simplification, the output of inverter V_S_ is a sinusoidal wave assuming a first harmonic approximation. Figure 2 shows the WPT Series-Series compensation network system, with source resistance R_S_ and load resistance R_L_ which is like a two port network. In general, applying KVL,

2 $$V = IZ$$

where, Z is the two-by-two impedance matrix for the dual-coil WPT system,

3 $$Z_{eq} = \left( {\begin{array}{*{20}c} {Z_{11} } & {Z_{12} } \\ {Z_{21} } & {Z_{22} } \\ \end{array} } \right)$$

Each element of the impedance matrix (Z_eq_) represents the impedance due to the self-inductance (L) and mutual inductance (M) between the other coils; the expressions for each element are given as follows. Here, M_12_ indicates the mutual inductance between the transmitter and receiver coil.

4 $$Z_{11} = R_{s} + R_{1} + j(\omega L_{1} - \frac{1}{{\omega C_{1} }}),\;Z_{12} = - j\omega M_{12}$$

5 $$Z_{21} = - j\omega M_{21} ,\;Z_{22} = R_{2} + j\left( {\omega L_{2} - \frac{1}{{\omega C_{2} }}} \right)$$

By substituting the above values to Eq. (3) matrix elements, the impedance matrix is obtained to determine the input and output power for the two-port network system.

6 $$Z_{eq} = \left( {\begin{array}{*{20}c} {R_{s} + R_{1} + j\left( {\omega L_{1} - \frac{1}{{\omega C_{1} }}} \right)} & { - j\omega M_{12} } \\ { - j\omega M_{21} } & {R_{2} + j\left( {\omega L_{2} - \frac{1}{{\omega C_{2} }}} \right)} \\ \end{array} } \right)$$

Using an impedance matrix (6), the Kirchhoff voltage law equation for a coil WPT system is obtained from Eq. (3) with transmitter coil excitation voltage V_S_

7 $$\left( {\begin{array}{*{20}c} {V_{S} } \\ 0 \\ \end{array} } \right) = \left( {\begin{array}{*{20}c} {I_{1} } \\ {I_{2} } \\ \end{array} } \right)\left( {\begin{array}{*{20}c} {R_{s} + R_{1} + j\left( {\omega L_{1} - \frac{1}{{\omega C_{1} }}} \right)} & { - j\omega M_{12} } \\ { - j\omega M_{21} } & {R_{2} + j\left( {\omega L_{2} - \frac{1}{{\omega C_{2} }}} \right) + R_{L} } \\ \end{array} } \right)$$

Mutual inductance for passive networks M_12_ = M_21_, M_23_ = M_32_, and M_13_ = M_31_, since Z-parameters are defined with either input open-circuited or output open-circuited. At resonance, inductive and capacitive reactance compensated each other; the PTE of the coil WPT system is given by,

8 $$\eta = \frac{{P_{out} }}{{P_{in} }}$$

From voltage matrix Eq. (7) voltage equation is derived for each coil of the two-coil WPT system,

9 $$V_{s} = I_{1} \left( {R_{1} + R_{S} } \right) - j\omega_{0} I_{2} M_{12}$$

10 $$0 = I_{2} \left( {R_{2} + R_{L} } \right) + j\omega_{0} I_{1} M_{21}$$

Solving the Eq. (9 and 10), then the expression for I_2_ is given by,

11 $$I_{2} = \frac{{j\omega_{0} M_{12} }}{{R_{2} + R_{L} }}I_{1}$$

To find out the input power of the two-coil WPT system network, expressions of I_2_ are substituted in Eq. (9),

12 $$V_{s} = \left[ {\left( {R_{1} + R_{s} } \right) + \frac{{\omega_{0}^{2} M_{21} M_{12} }}{{R_{2} + R_{L} }}} \right]I_{1}$$

The total input power of the Wireless Power Transfer (WPT) system, a two-port network, is determined by multiplying the source voltage input by the input current of the two-port network.

13 $$P_{in} = V_{s} *I_{1}$$

Simplified source input voltage expression (12) is substituted in the input power Eq. (13), which gives,

14 $$P_{in} = \left[ {\left( {R_{1} + R_{s} } \right) + \frac{{\omega_{0}^{2} M_{21} M_{12} }}{{R_{2} + R_{L} }}} \right]I_{1}^{2}$$

The total output power is found by using the following equation,

15 $$P_{out} = I_{2}^{2} R_{L}$$

where I2 is the load current of the network, the simplified expression for I2 is derived in Eq. (11). Now output power is derived as follows,

16 $$P_{out} = \frac{{j\omega_{0} M_{12} R_{L} }}{{R_{2} + R_{L} }}I_{1}^{2}$$

Substitute Eqs. (15 and 16) in the expression for the wireless power transfer efficiency Eq. (8).

17 $$\eta = \frac{{R_{L} }}{{\left( {R_{1} + R_{s} } \right)\left( {\frac{{R_{2} + R_{L} }}{{\omega_{0} M_{21} }}} \right)^{2} + \left( {R_{2} + R_{L} } \right)}}$$

Since, $$\omega_{0} > > \left( {{\text{R}}_{{2}} + {\text{R}}_{{\text{L}}} } \right)$$ therefore,

18 $$\eta = \frac{{R_{L} }}{{R_{2} + R_{L} }}$$

The efficiency is maximized if the R_2_/R_L_ value is kept minimum.

The resistance R_L_, which is commonly referred to as the corresponding load resistance, from Eq. (18) the maximum power can be transferred if load resistance is made higher than receiver coil resistance (R_2_).

### CC and CV modes battery charging

**Figure 3:**
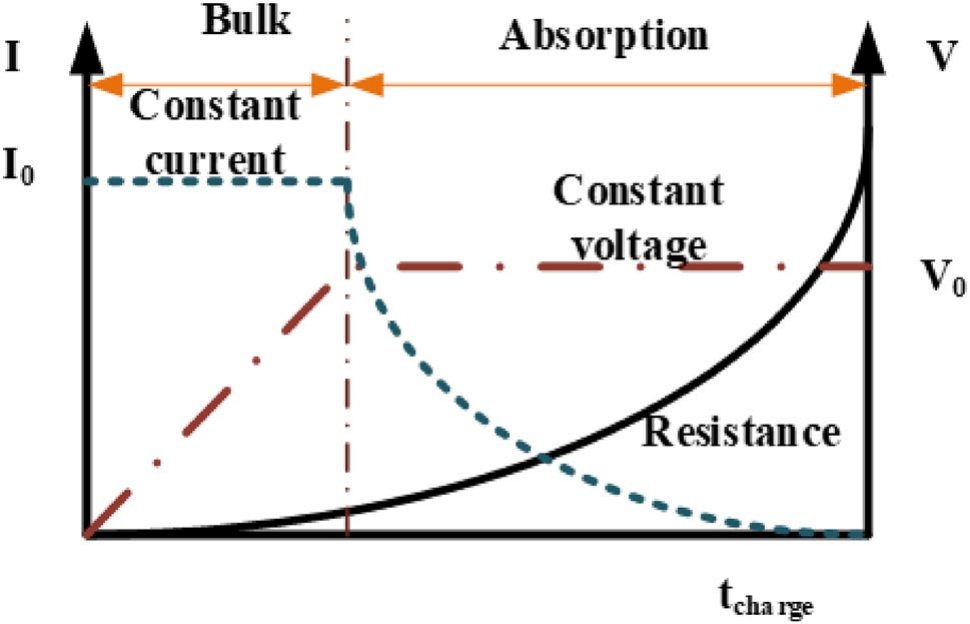
CC/CV Charging.

To prolong the lifespan of lithium-ion batteries, a two-phase charging method called CC/CV (Constant Current/Constant Voltage) is utilized^26^. This approach entails charging the battery at a constant current until it attains the rated or nominal battery voltage specified by the manufacturer. Upon reaching this threshold, the charging process transitions to maintaining a constant voltage^27,28^. The selection of the charging current value is influenced by the properties of the battery, and opting for values below the suggested maximum can help prolong the battery's lifespan. The primary objective of this research is to assess the constructed model and investigate the impact of obstructive substances on a thorough Constant Current-Constant Voltage (CC-CV) charge, as seen in Fig. 3.

Various methodologies have been employed in the literature to implement this control mechanism for wireless charging of generic Electric Vehicles (EVs). The specific approach adopted often relies on the converter used and the communication systems integrated into the design. Notably, the proposal outlined in^29^ modifies the topology by incorporating switches for switching between devices operating in each phase.

Nevertheless, a significant limitation of this approach is its incapacity to regulate charging current or voltage, presenting challenges in achieving compatibility among different devices. In the domain of power electronics design, this proposal is associated with additional drawbacks, such as heightened design complexity and an increased number of components. Consequently, it results in elevated costs and weight in a system striving to minimize both.

The SS Compensation network is typically configured as the main compensation method for IPT systems because it is straightforward to maintain a constant current in the primary coil^30^. When the secondary coil is connected, a reliable induced voltage is produced on the secondary side. Consequently, the SS topology is commonly employed for CV mode. The resonance conditions for achieving Constant Current (CC) mode in the proposed system utilize Series-Series (SS) compensation due to its straightforward structure and voltage step-down characteristics when compared with Single-Parallel (SP), Parallel-Series (PS), and Parallel-Parallel (PP) compensation topologies. An equivalent circuit of the SS-compensated Inductive Power Transfer (IPT) system is depicted in Fig. 2.

In Eqs. (9 and 11), the voltage gain can be determined by the following manner. Furthermore, the symbol ω denotes the angular frequency, which is equal to 2π multiplied by the regular frequency f.

The voltage gain derived from the aforementioned equation is expressed as follows:

19 $${G}_{V}=\frac{{V}_{out}}{{V}_{S}}=\frac{\omega M}{{Z}_{1}{Z}_{2}+{\omega }^{2}{M}^{2}}*{R}_{L}$$

where V_s_ is the input voltage at the primary side, V_out_ is the output voltage at the secondary side, and M is the mutual inductance of L_1_ and L_2_. Considering the presence of a rectifier with a capacitor filter, acting as an impedance transformer, R_L_ can be adjusted as an equivalent AC resistance R_L – AC_. The load resistance can be substituted with R_L − AC_, represented in terms of the battery voltage V_battery_ and the charge current I_charging_, as shown in Eq. 20.

20 $$R_{{{\text{L}} - {\text{A C}}}} = \frac{8}{{\pi^{2} }}R_{L} = \frac{8}{{\pi^{2} }}\frac{{V_{{{\text{battery}}}} }}{{I_{{{\text{charging}}}} }}$$

Based on Eqs. (19 and 20), the frequency can be adjusted to deliver a constant current (CC) to the battery. The mutual inductance and voltage are influenced by the coil arrangement and battery characteristics. The charging current is predetermined based on the recommended charge current rating for the battery. The optimal frequency can be determined from the following equation:

21 $$V{V}_{\text{Gain}\_\text{Diff}}={V}_{\text{Gain}\_\text{Frequency}}-{V}_{\text{Gain}\_\text{Voltage}}$$

22 $${Voltage}_{\text{Gain}\_\text{Diff}}={V}_{\text{battery}}\left\{\left|\frac{\omega M}{{\pi }^{2}({Z}_{p}{Z}_{s}+{\omega }^{2}{M}^{2}){I}_{\text{charging}}}\right|-\frac{1}{{V}_{\text{in}}}\right\}$$

**Figure 4:**
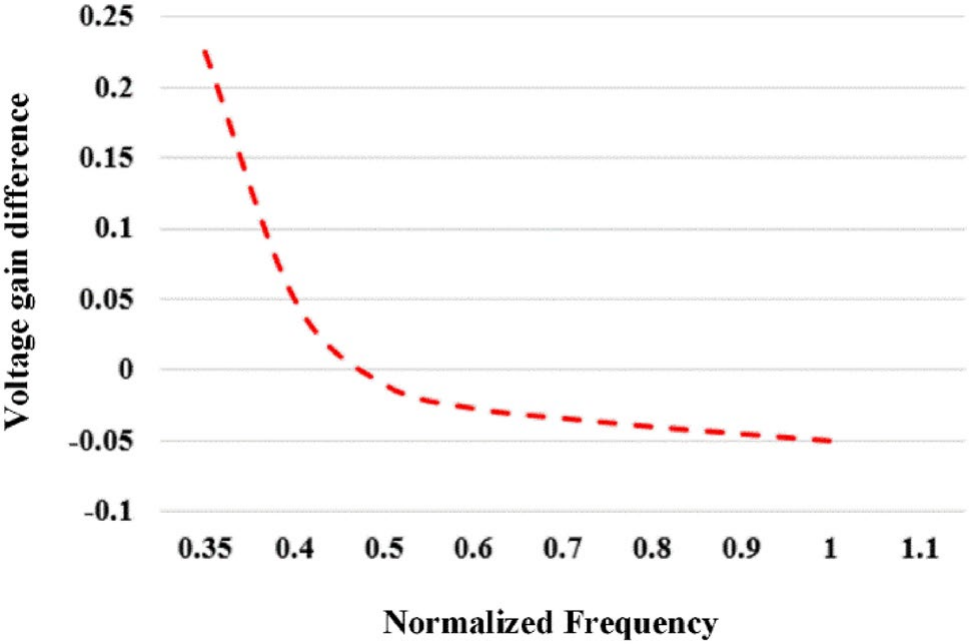
Voltage gain Vs normalized frequency voltage and current at ratted condition.

V_Gain_diff_ denotes the disparity in voltage gain between the frequency-dependent gain (V_Gain_freq_) and the target gain (V_Gain_voltage_). The target gain is established based on the current battery voltage and charging current, as outlined in Fig. 4, by the specified battery characteristics.

In Fig. 4, the plot illustrates the voltage gain difference (V_Gain_diff_) about the normalized frequency across different voltage and current scenarios. When V_Gain_diff_ reaches zero, the control frequency for the desired battery current and voltage can be set. Determining this control frequency involves employing the interpolation method.

**Figure 5:**
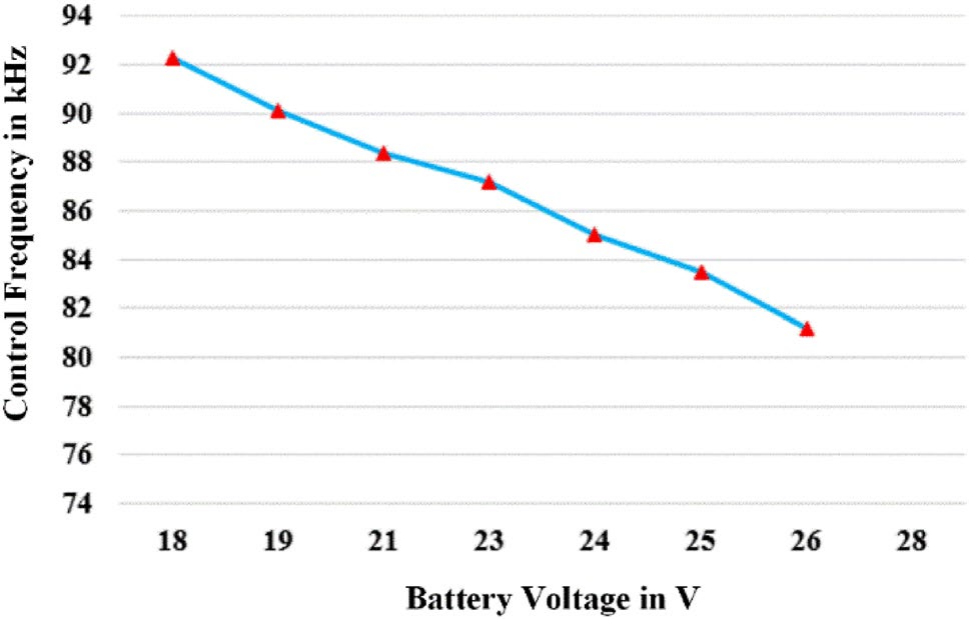
Charging Frequency Vs Battery Voltage.

Below the resonance frequency, the frequency falls into the capacitive region, where the current leads. Within this range, there is a risk of shoot-through current at the diode of the half-bridge inverter switch. To address this issue, frequency modulation is utilized in the region above the resonance frequency. Figure 5 provides a depiction of the charging frequency of the battery voltage. The calculated frequency is determined using Eq. (19), assuming a constant input voltage and a battery charging current of 2 A.

Figure 3, provided alongside, portrays the theoretical implementation of CC/CV charging for the battery utilized in the prototype. The procedure entails an initial stage of constant current at 2 A, wherein the voltage increases, succeeded by a constant-voltage stage at 24 V, where the charging current gradually diminishes. Crucially, ensuring compatibility with other bikes is accomplished by utilizing the battery characteristics provided by the second controller for charging references and the selector's determination. In the case of our particular e-bike, the values for V_ref_ and I_ref_ are established at 24 V and 2 A, respectively.

## Implementation of WPT on EV

A WPT-based EV charging system can address multiple factors, including the selection of WPT technology, design of EV for WPT, Battery for WPT Vehicle, Design of WPT Coils, Design of tracking system and power transfer efficiency.

### Structure of EV for WPT

**Figure 6:**
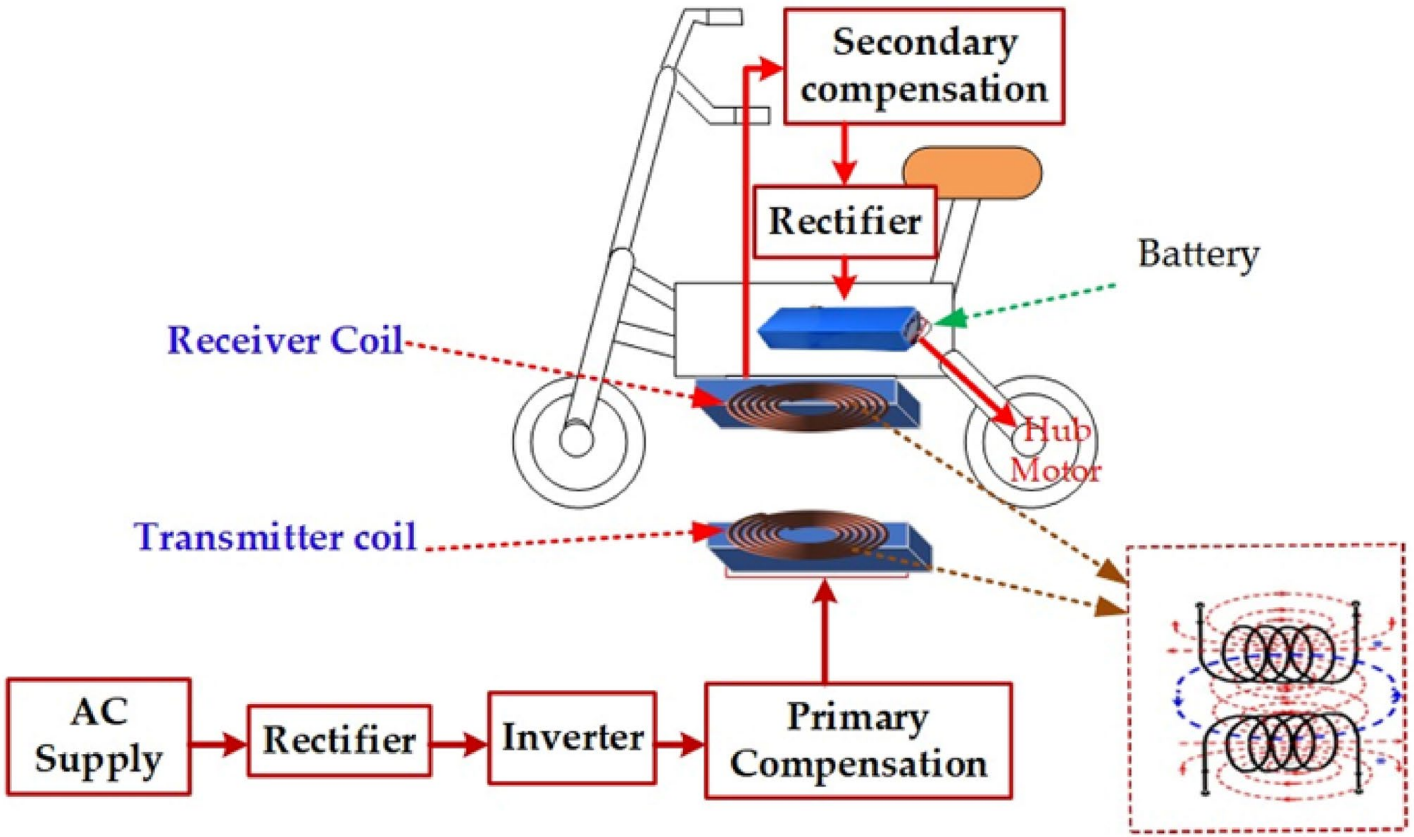
EV that Integrates WPT Technology.

Transmitter generates an alternating magnetic field captured by the receiver on the EV. The magnetic field is subsequently transformed into electrical energy via the receiver, which is utilized to recharge the battery pack. The presence of misalignments between the transmitting and receiving coils has a detrimental impact on the coupling and efficiency of power transfer^31^. It is necessary to align transmitter and receiver coil at same vertical plane to maximize the capability of power transfer from grid to battery. Such improper alignment increases the charging time and cause over heating in the coils and resonant components, which decreases transfer efficiency and its lifespan. Figure 6. Depict the setup of an EV with wireless charging.

Therefore, in WPT based EV charging system, the primary concern for the improvement of power transfer efficiency is the proper alignment of the transmitter and receiver coil. Particularly in the case of circular type coils the flux distribution is at its maximum at center of the pad. Due to any lateral or angular misalignment the flux linkage gets affected further which reduces the coupling between transmitter and receiver pad. This increases the search for investigations to eliminate or minimize the misalignment in WPT-based EV charging system.

### Design of EV for WPT

In the context of the schematic depiction of Wireless Power Transfer for electric vehicles, the development of the electric vehicle aligns with a suitable EV model. The first phase in modelling the vehicle's performance entails formulating an equation for the necessary tractive effort. This term denotes the force responsible for propelling the vehicle forward, transmitting it to the ground through the drive wheels^32^.

Rolling resistance primarily results from hysteresis losses within the vehicle tire. Furthermore, friction within the gearing system and bearings contributes to this resistance. Unlike other resistances, rolling resistance stays relatively constant and is largely unaffected by the vehicle's speed^33^. Instead, it is directly proportional to the weight of the vehicle. The equation that represents rolling resistance is as follows

23 $${\text{F}}_{{{\text{rr}}}} = \mu_{{{\text{rr}}}} {\text{mg}}$$

where m is mass, μrr is the rolling resistance coefficient and g is the gravitational constant.

Aerodynamic drag refers to the resistance a moving object encounters when travelling through the air at a specific velocity. The aerodynamic drag of an object is influenced by several elements, including its frontal area, shape, and any protrusions it possesses, such as side mirrors, ducts, air passageways, and spoilers. These elements jointly determine the extent of aerodynamic drag. The calculation of this component is determined by the following formula^34^.

24 $${\text{F}}_{{{\text{ad}}}} = {\text{12C}}_{{\text{d}}} {\text{A}}\rho {\text{V}}^{{2}}$$

C_d_ represents the drag coefficient. The symbol ρ represents the density of air. Determining the force necessary to propel a vehicle uphill is a pretty simple process. It involves calculating the portion of the vehicle's weight that is aligned with the slope. The magnitude of this force is directly determined by the steepness of the slope and the mass of the vehicle. Through the analysis of these variables, one can determine the amount of force required to propel the vehicle upward.

25 $${\text{F}}_{{{\text{hc}}}} = {\text{mgsin}}\Psi$$

When the velocity of a vehicle changes, it is apparent that some extra force must be exerted to explain the acceleration. This force is accountable for the straight-line acceleration of the vehicle and is defined by a widely recognized equation derived from Newton's third law.

26 $${\text{F}}_{{{\text{la}}}} = {\text{ma}}$$

For Angular Acceleration,

27 $${\text{F}}_{{{\text{wa}}}} = {\text{I G}}^{{2}} /\left( {\eta {\text{g r}}^{{2}} } \right){\text{ a}}$$

where G is the gear ratio and ηg is the gear system efficiency.

The total tractive effort is given by,

28 $${\text{F}}_{{{\text{te}}}} = {\text{F}}_{{{\text{rr}}}} + {\text{F}}_{{{\text{ad}}}} + {\text{F}}_{{{\text{hc}}}} + {\text{F}}_{{{\text{la}}}} + {\text{F}}_{{\omega {\text{a}}}}$$

where F_rr_ is the rolling resistance force,

F_hc_ is the hill climbing force,

F_la_ is the force required to give linear acceleration,

F_ad_ is the aerodynamic drag,

F_ωa_ the angular acceleration force.

Required power is given by,

29 $$\mathbf{P}\mathbf{o}\mathbf{w}\mathbf{e}\mathbf{r}=\mathbf{T}\mathbf{o}\mathbf{t}\mathbf{a}\mathbf{l}\mathbf{t}\mathbf{r}\mathbf{a}\mathbf{c}\mathbf{t}\mathbf{i}\mathbf{v}\mathbf{e}\mathbf{f}\mathbf{o}\mathbf{r}\mathbf{c}\mathbf{e}\times \mathbf{v}$$

30 $$\mathbf{P}={\mathbf{F}}_{\mathbf{t}\mathbf{e}}\times \mathbf{v}$$

where ‘v’ is the velocity of the vehicle in m/s.

Table 1 shows all the electrical and mechanical parameters for the design of EVs. All the parameters implemented in Table 1 have been considered and implemented in the design of EVs.

**Table 1:** Parameters considered for the design of EV.

Parameters	Notation	Vehicle	
		Value	Units
Weight (Vehicle + commuter)	M	150.00	Kg
Width	W	0.6	m
Height	H	1.00	m
Air Density	$\rho$	1.25	Kg/m^2^
Rolling Resistance	$\mu$	0.02	–
Drag Coefficient	C_d_	0.70	–
Gravitational Force	g	9.8	–
Speed in km	S	40.00	Kmph
Frontal Area	A	0.6	m^2^
Velocity	v	11.11	m/sec
Angle – Hill climbing	$\theta$	–	Degree
Rolling Resistance Force	F_RR_	23.32	Watts
Aerodynamics Drag Force	F_ad_	10.56	Watts
Hill Climbing Force		–	Watts
Tractive Force	F_te_	34.56	F_RR_ + F_ad_
Total Power for 40 km	P	384	Watts

### Battery for WPT vehicle

The designed vehicle is implemented with a lithium-ion battery. In this section, the design for a battery pack for the 40 km range and the travel factor will be considered. The total range is considered 40 km and the total speed is 40 kmph.

31 $$\text{Travel factor}=\frac{\text{Total Range}\left(\text{Travel}\right)}{\text{Total Speed}}=\frac{40}{40}=1$$

This travel factor is to travel 40 km at 40 kmph speed constantly. So, the power required will be,

32 $$Power \times Travel factor = 384 \times 1 = 384 Wh$$

So, the total power required will be 384 Wh. Then, the efficiency of the battery pack has to be considered. Lithium–ion batteries usually have 85%—93% charging and discharging efficiency. Considering the efficiency as 85%,

33 $$\text{Battery pack capacity required}=\frac{\text{Total Power}}{\text{Efficiency}}=\frac{384}{0.85}=451.74\text{ Wh}$$

### Design of WPT coils

This section is dedicated to designing a circular coil pair for a mid-range medium-power wireless charging system capable of delivering up to 350W. Achieving optimal performance necessitates modelling the coil pair based on the configuration of a coreless transformer. Failure to incorporate a ferrite core may result in substantial inductance leakage, leading to additional losses. To address this concern, Resonant Inductive Wireless Power Transfer (RIWPT) can be utilized, thereby improving power transfer efficiency and enhancing mutual inductance.

34 $$\sum {\text{B}}_{\text{T}}\Delta \text{l}= {\upmu }_{0}{\text{IN}}_{\text{P}}$$

35 $${\text{E}} = - {\text{N}}_{{\text{S}}} \frac{{{\text{d}}\phi_{{\text{B}}} }}{{{\text{dt}}}}$$

where, ${B}_{T}$ is the magnetic flux density,

${\mu }_{0}$ is the permeability of free space,

$\Delta l$ is the length of the conductor,

E is the Induced voltage,

${\varnothing }_{B}$ is the magnetic flux,

${N}_{P}$ and ${N}_{S}$ represents several primary and secondary turns.

### Self-inductance of coils

In the context of coil design, D_out_ refers to the coil's outer diameter, while D_in_ represents its inner diameter. S denotes the distance between turns, and w signifies the wire's diameter. The inductance of spiral coils can be calculated using Eq. (36), which is based on the original Wheeler expression.

36 $${\text{L}} = {\text{a}}^{2} {\text{N}}^{2} \mu {\text{H}}$$

Which can be used to determine the inductance value in µH. This equation incorporates the number of turns denoted by 'N'.

37 $$\text{c}=\frac{ {\text{d}}_{\text{out}}- {\text{d}}_{\text{in}}}{2}$$

38 $$\text{a}= \frac{{\text{d}}_{\text{out}}-C}{2}$$

A simplified and more practical version of the Wheeler formula can be created by incorporating (37) and (38) in (36) as illustrated in (39)

39 $$\text{L}= \frac{{\text{N}}^{2}({{\text{D}}_{\text{out}}+{\text{D}}_{\text{in}})}^{2}}{8\left(15{\text{D}}_{\text{out}}-7{\text{D}}_{\text{in}}\right)2.54}$$

When all the dimensions are measured in centimeters, Eq. (39) provides the inductance value in µH. The author make use of this modified Wheeler formula for coil design. Equation 42 explains the relationship between D_out_ and D_in._

Equations 42 and 43 allow for the determination of all the geometric characteristics of coils based on the provided value of inductance. The initial step in the coil design process involves selecting a value for D_out_. One approach to determining D_out_ is by considering the air gap required between the primary and secondary coils.

### Design of electrical parameters for coils

Calculations of the electrical parameters were made for a 24 V output at 350 W and a resonance frequency of 85 kHz. The assumed input voltage was 48 V. V_rms_ and was maintained at 26 V rather than Vo because of the wire's constrained ability to transmit current. The electrical parameters are determined using the methods described in this section, and are as follows:

40 $${\text{R}}_{0}=\frac{{{\text{V}}^{2}}_{\text{rms}}}{{\text{P}}_{0}}=\frac{{\left(26\right)}^{2}}{350}=1.04$$

$${\text{R}}_{\text{LR}}=\frac{8}{{\uppi }^{2}}\times 1.04=0.84\Omega ,{\text{R}}_{\text{LB}}=\frac{{26}^{2}}{350}=1.93\Omega$$

41 $${\text{R}}_{\text{L}}={R}_{LR}+{R}_{LB}=0.84+1.93=2.77\Omega$$

42 $${\text{V}}_{\text{Srms}}=2\sqrt{2}\times \frac{{\text{V}}_{0}}{\uppi }=2\sqrt{2}\times \frac{26}{\uppi }=23.4$$

43 $${\text{I}}_{\text{Srms}}=\frac{{\text{V}}_{\text{Srms}}}{2.77}=\frac{23.4}{2.77}=8.45\text{ A}$$

As a Quality factor, Q_s_ is considered to be 4 and k = 0.2,

44 $${\text{L}}_{{\text{S}}} = \frac{{{\text{Q}}_{{\text{S}}} {\text{R}}\_{\text{L}}}}{{\omega_{0} }} = \frac{4 \times 1.04}{{2 \times \pi \times 85000}} = 6.29{ }\mu {\text{H}}$$

45 $${\text{I}}_{\text{Prms}}=\frac{350}{43.2}=8.1\text{ A}$$

46 $${\text{M}} = \frac{{{\text{I}}_{{{\text{Srms}}}} \times {\text{R}}_{{\text{L}}} }}{{{\text{I}}_{{{\text{Prms}}}} \times \omega_{0} { }}} = \frac{8.45 \times 2.77}{{8.1 \times 2 \times \pi { }85000{ }}} = 5.4{ }\mu {\text{H}}$$

47 $${\text{L}}_{{\text{P}}} = \frac{{{\text{M}}^{2} }}{{{\text{L}}_{{\text{S}}} \times {\text{k}}^{2} }} = \frac{5.4 \times 5.4}{{6.29 \times 0.2 \times 0.2}} = 115.9{ }\mu {\text{H}}$$

The values for the compensation capacitors can be calculated using a typical empirical formula associated with resonant circuit. All the calculated circuit and electrical parameters are shown in Table 2. From the inductance values of the primary (L_p_) and secondary (L_s_) coil, the number of turns and, the inner and outer diameter of the coil can be calculated. Then, from the capacitance values, the resonant frequency can be calculated.

**Table 2:** Calculated parameters for 350 W setup.

Parameters	Values
V_Srms_	23.4 V
I_Srms_	8.45 A
V_Prms_	43.2 V
I_Prms_	8.1 A
R_L_	2.77 Ω
L_P_	115.9 µH
L_S_	6.29 µH
M	5.40 µH
C_P_	48 × 10^–4^ µF
C_S_	569 × 10^–4^ µF

The complete description of the EV design the design of the battery pack and the calculated parameters for design were tabulated. The chapter also provides the coil design calculation for finding the self-inductance of coils and the calculation for the electrical parameters of 350 W for WPT. It also provides the standard adhered to for the design of WPT.

### Tracking system control using proposed sensing coil

**Figure 7:**
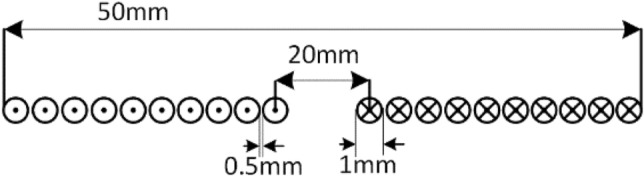
Sensing Coil parameters.

In general, mainly two different types of misalignment are prominent in case of circular pads, which are lateral and angular misalignment. In the considered application the EV is always parked in the prescribed position, therefore the centers of the transmitter and receiver coil is always aligned each other. Possibilities of the lateral misalignment is less; angular misalignment needs to be improved. Two micro circular sensing coils (S_1_ & S_2_) were employed to determine the alignment of the receiver pad. The dimensions of the sensing coil are shown in the Fig. 7, the self-inductance of the sensing coil is 4µH. The sensing coil is placed over the transmitter pad as illustrated.

The mutual inductance between transmitter and sensing coil-1 is M_S1T_, mutual inductance between receiver coil and sensing coil-1 is M_S1R_, likewise for sensing coil-2 are M_S2T_ & M_S2R_. When the transmitter coil is excited, circulates current in both transmitter and receiver coil. Due to the angular misalignment the mutual inductance between sensing coils and receiver coil varies. This variation in the mutual inductance is mainly depends on the vertical distance between receiver coil and sensing coil. Based on the variation of the mutual inductance values the transmitter pad is aligned coaxially with receiver pad.

**Figure 8:**
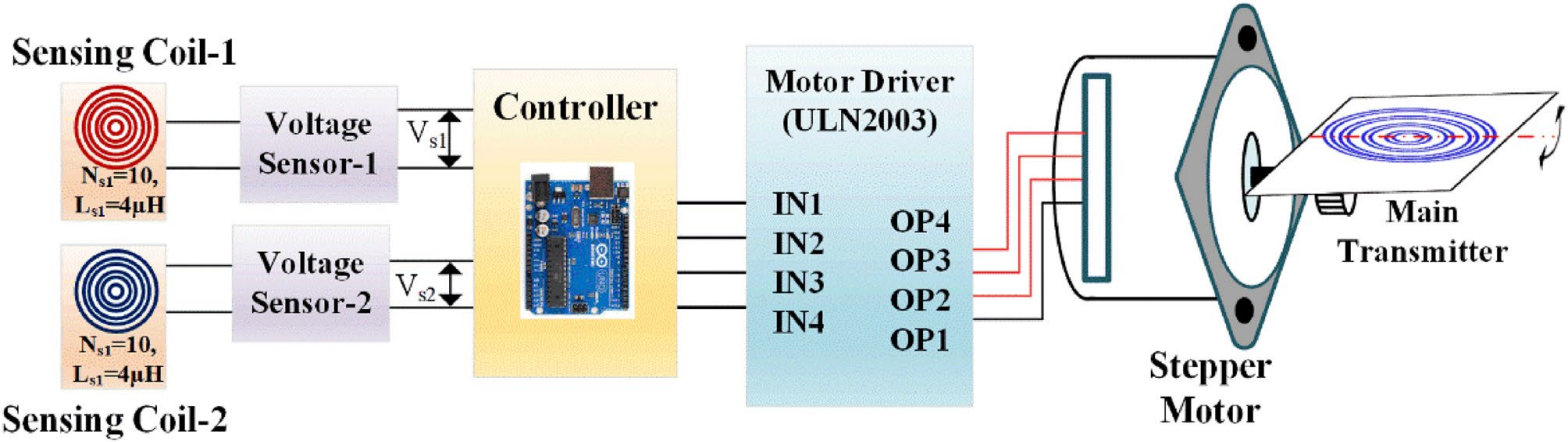
Schematic diagram of proposed transmitter coil aligning system.

The schematic diagram shown in Fig. 8 illustrates the proposed coil aligning mechanism. It uses additional sensing coil of 10 turns, its physical dimensions shown in Fig. 8 for identifying and positioning the transmitter along with receiver coil. The transmitter coil is placed over the mounting plate, which is arranged with axial rotation by connecting the center axis with pulley at one end and another end with motor’s shaft. The stepper motor is powered with its compatible driver (ULN2003) operated by Arduino microcontroller. Voltage sensors gives the input control signal to microcontroller, the received signal strength is depending on the magnetic flux strength. The controller sets the command to the motor to rotate clockwise or anti-clockwise based on the logical difference between the received signals.

This alignment operation is primarily relying on the axis control algorithm, specifically designed for this wireless EV charging tracking system. In this section the entire operation of the proposed algorithm is described. This algorithm plays a crucial role in aligning the transmitter coil with the receiver coil to transfer maximum possible power. Its distinct and intricate nature necessitates keen attention and comprehension to ensure the efficient operation of the charging device. This section will thoroughly examine the inner mechanisms of the algorithm, delving into its complexities and subtleties to achieve a thorough grasp of its capabilities and constraints. Through a comprehensive analysis, this section aims to elucidate the workings, potential, and limitations of the algorithm while emphasizing its significance in the realm of wireless charging for electric vehicles.

It is created to be simple, following a test-driven and agile approach, even without using test harnesses alongside the Arduino IDE. This simplicity is beneficial for the product’s goals and expectations at its current development stage. The algorithm aims to achieve autonomous alignment and uses a basic single-axis approach tested in a lab setting. When activated, it instructs the motors to drive clockwise or anti-clockwise until a set point value from the sensor is reached.

**Figure 9:**
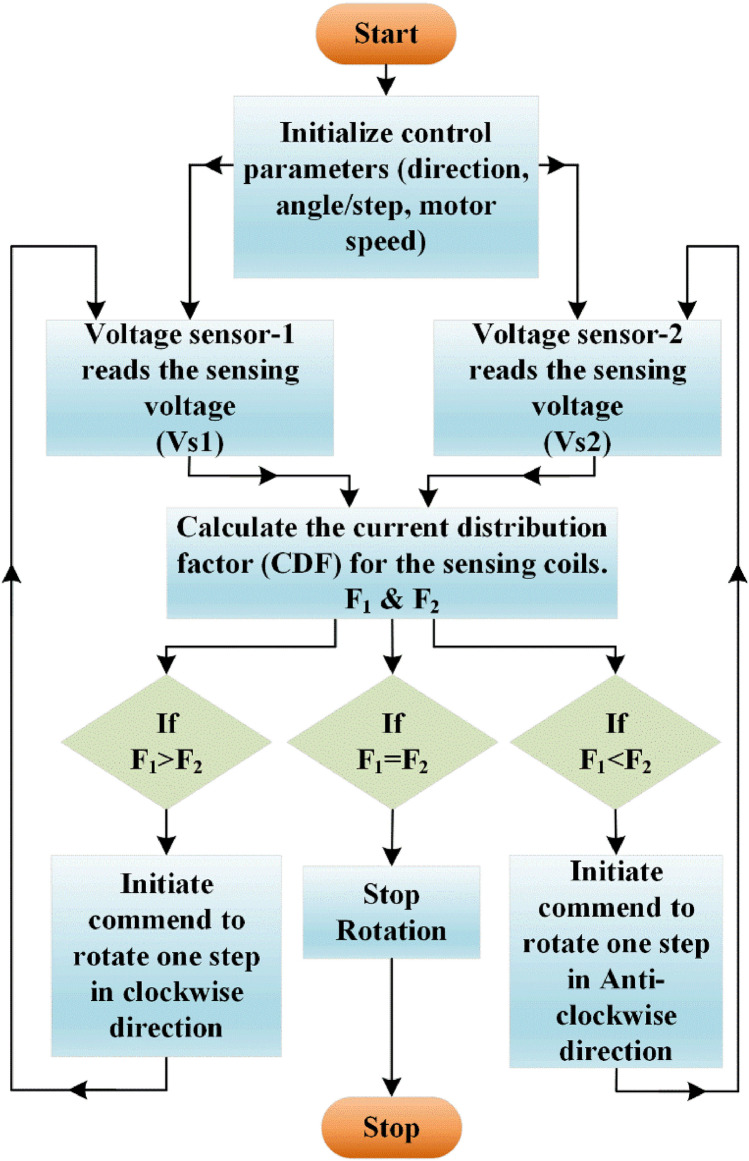
Flowchart of the axis control algorithm.

Figure 9 shows the flow chart for the proposed algorithm, single stepper motor is used to control the axis of the coil mounting plate. After initializing the basic required parameters of the stepper motor, the process starts from identifying the position of the receiver coil based the sensors output. Current distribution factor (CDF) is calculated for each sensing coils (F_1_ & F_2_) using Eq. (53), by comparing this CDFs control Commend is generated to rotate the motor towards clockwise or anticlockwise direction until the perfect alignment between transmitter and receiver is reached.

**Figure 10:**
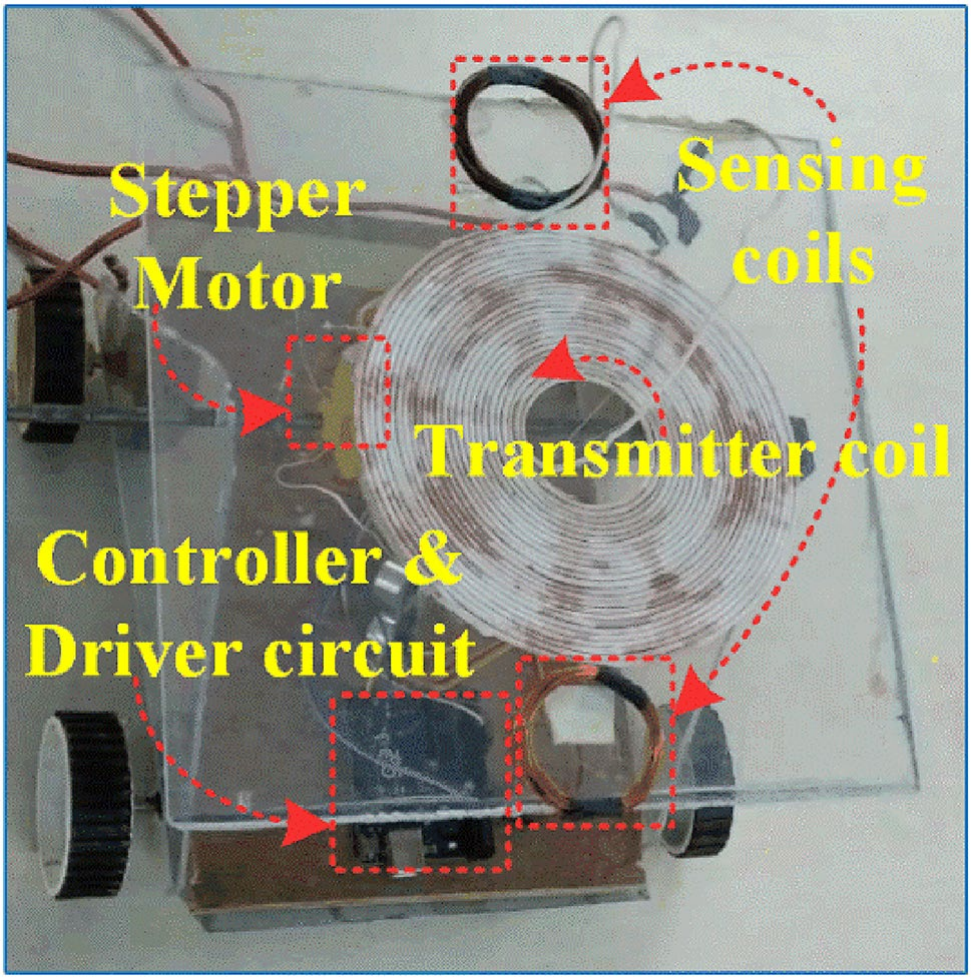
Entire arrangement of the transmitter coil alignment system.

The arduino microcontroller directs the motor drive shield to operate the motors according to the programmed algorithm. With its ample capacity, the arduino can easily handle the instructions and has room for future enhancements like additional sensors. The sensing coils were positioned at top of the alignment system as shown in the Fig. 10, its output is processed by the microcontroller and commends the motor. Such arrangement accurately captures the position of the receiver coil and makes the positioning of the transmitter coil. For convenient positioning of the transmitter the entire alignment system along with stepper motor is fixed over the movable chase.

The tracking system is validated with the designed prototype EV of capacity 350W. Selection of optimum number of sensing coil is primarily based on the application. In case of two wheelers the possibilities of the angular misalignment over front and back side is minimum unless the parking surface is not flat. Possibility of angular misalignment over adjacent is prominent, therefore two sensing coils is placed over the adjacent sides of the transmitter pad, as shown in Fig. 10.

The magnetic field strength is accurately sensed by calculating the CDF value of the each coil. From Eq. 48, the CDF is obtained, this CDF is used for the identification of receiver coil with ‘k’ number of transmitter coils. Here CDF is used for the identification of receiver coils position with ‘k’ as number of sensing coil.

48 $$F_{k = 1,2,3...} = \frac{1}{{\sqrt {\sum\limits_{l = 1}^{\infty } {\left( {\frac{{M_{kr} }}{{M_{lr} }}} \right)}^{2} } }}$$

Based on the general equation for finding the CDF the CDFs for the proposed sensing coil were obtained as given in the equation (49) & (50)

49 $$F_{1} = \frac{1}{{\sqrt {1 + \left( {\frac{{M_{S1r} }}{{M_{S2r} }}} \right)^{2} } }}$$

50 $$F_{2} = \frac{1}{{\sqrt {1 + \left( {\frac{{M_{S2r} }}{{M_{S1r} }}} \right)^{2} } }}$$

where F_1_ and F_2_are CDFs of sensing coils 1 and 2 respectively. Therefore, it is clear from equations 49, 50 finding the ratios of mutual inductances between sensing coils and Rx coil is necessary to find CDF. When transmitter coil is stimulated with an external source, which induces the EMF in the Rx coil and sensing coils. The following expressions give the induced voltage across the sensing coil.

51 $$V_{1} = j\omega M_{1r} I_{r}$$

52 $$V_{2} = j\omega M_{2r} I_{r}$$

I_r_ is the Rx current when Tx coil is excited, and V_1_ & V_2_ are induced voltage across sensing coils. The mutual inductance ratio is calculated from equations 51 & 52.

53 $$\frac{{V_{1} }}{{V_{2} }} = \frac{{M_{S1r} }}{{M_{S2r} }}$$

Using the above mutual inductance ratios (53), CDFs are calculated, which can be used to detect the position of Rx coil's.

**Figure 11:**
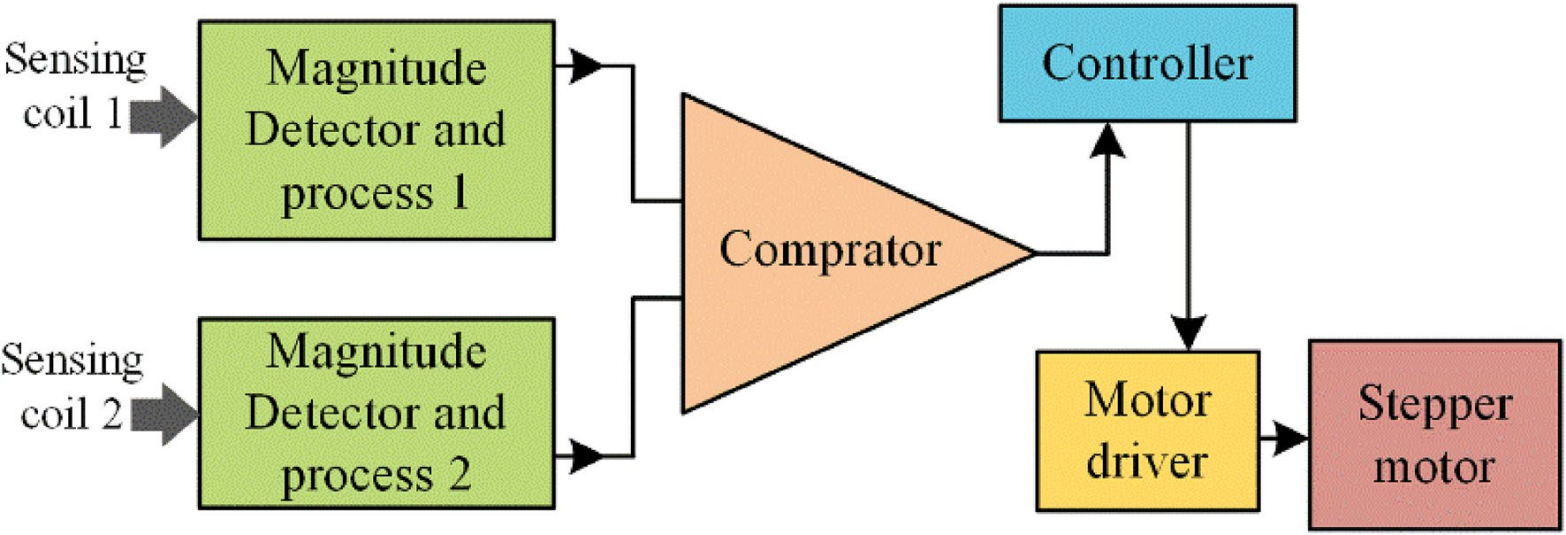
Block diagram of tracking control over the transmitter pad.

Stepper motor is used to tilting the transmitter pad based on the controller output through the driving circuit block diagram as shown in Figure 11. The CDF factor were calculated by the magnitude block, then which is compared with reference input using comparators. The CDFs of the two sensing coil is compared and error is given to the PI controller. The output of the PI controller controls the stepper motor driving rotation with driver circuit. Thus the sensing coil maintains the coaxial alignment of the transmitter pad with receiver pad.

## Simulation of coils using Ansys Maxwell

**Figure 12:**
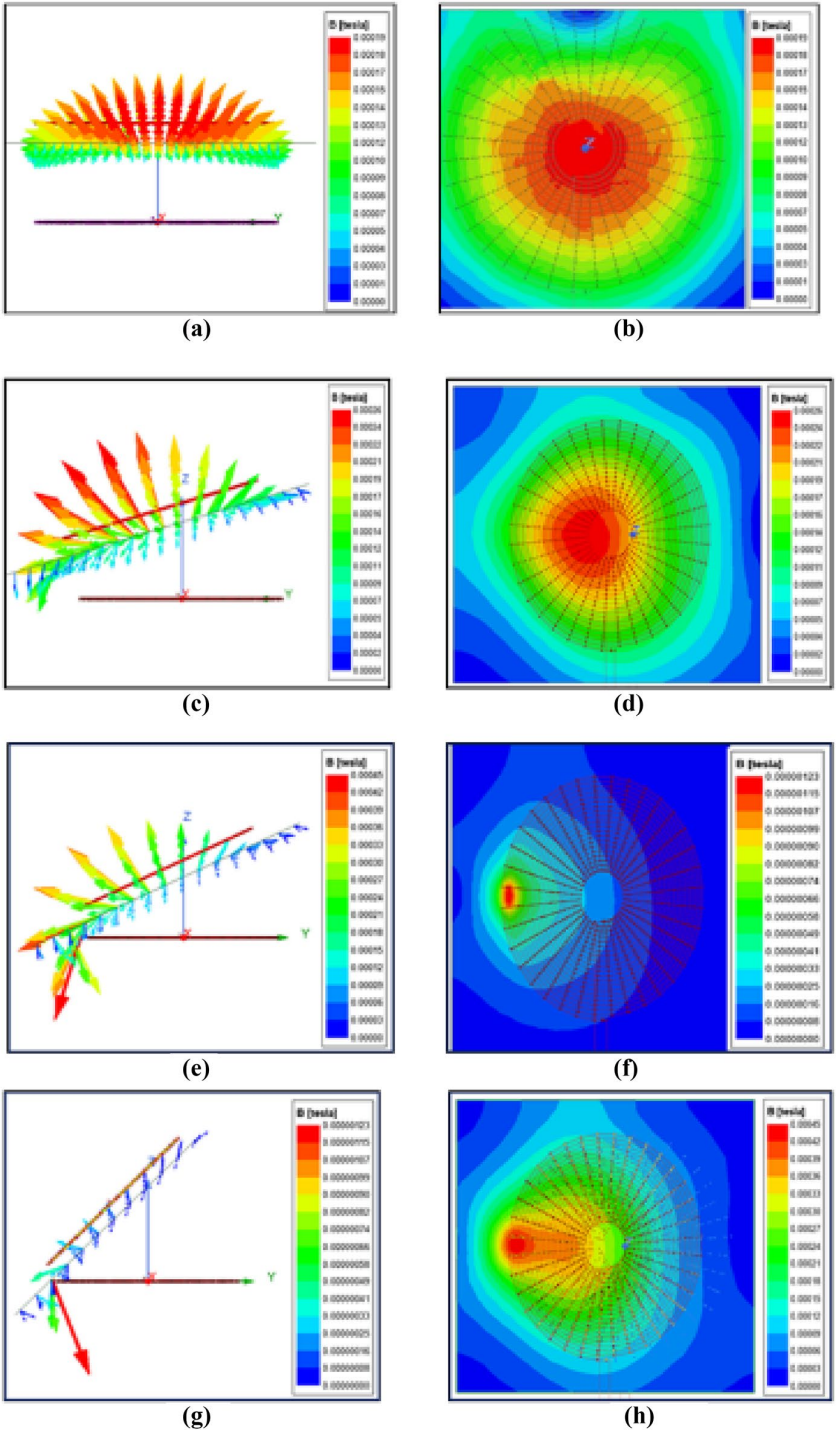
Magnetic flux distribution between coupling pads with angular misalignment of (**a**) 0°, (**b**)15°, (**c**)25°, (**d**)45° (Without tracking system).

Ansys Maxwell simulation software was utilized to model the coils in Fig. 12 during the examination of a wireless charger for an electric vehicle that is capable of accommodating misalignment. This software enables us to specify the characteristics of the coils, such as the number of turns, inner and outer diameter, as well as additional factors like wire size, insulation thickness, and material attributes. To simulate the coils, the magneto static analysis type is typically used. This type of analysis allows the calculation of the magnetic field distribution and other related quantities based on the steady-state condition. From this software, plotting the magnetic field strength along specific paths can be visualized, and the current density distribution in the coils can be calculated. Each coil in the model has 25 turns, a diameter of 20 cm, and a thickness of 2 mm.

The magnetic field intensity distribution during angular misalignment at different angles of the magnetic coupler is illustrated in Fig. 12. From Fig. 12a, the magnetic flux is equally distributed and focused on the middle of the coil. Unlike Fig. 12a,c–g show that the magnetic flux is not distributed or focused along the middle of the coil for various angles of misalignment. This leads to a reduction in mutual2inductance between the coils and, consequently, reduces the overall system efficiency. In Fig. 12c–h, the receiver coil is aligned at 15 degrees, 25 degrees, and 45 degrees, respectively, to that of the transmitter coil. The reduced mutual inductance for misalignment angles of 15 degrees, 25 degrees, and 45 degrees is 59.24T, 57.59T, and 53.33T, respectively.

Table 3 demonstrates a reduction in the mutual inductance of the Wireless Power Transfer (WPT) system as the angular misalignment between the coils increases. The gradual decrease in mutual inductance between the coils with increasing misalignment is also apparent. This reinforces the idea that a decrease in mutual inductance can lead to a reduction in the efficiency of the system.

**Table 3:** Variation of mutual inductance concerning angular misalignment.

S.no.	Misalignment angle(in degrees)	Mutual inductance (in µH)
1	0	64.74
2	15	59.24
3	25	57.59
4	45	49.23

**Figure 13:**
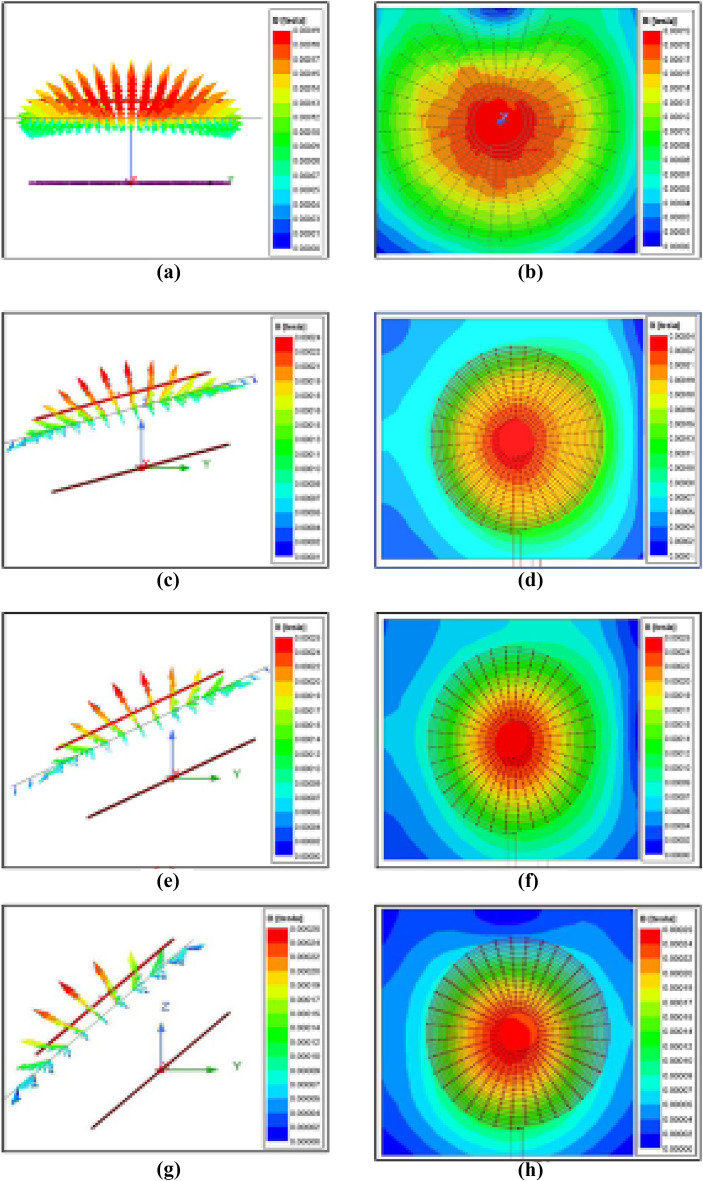
Magnetic flux distribution between coupling pads with angular misalignment of (**a**) 0°, (**b**) 15°, (**c**) 25°, (**d**) 45° (With tracking system).

Figure 13 depicts the simulation where the coils are not misaligned, resulting in no change in flux. In contrast, Figs. 13c–h show scenarios where the transmitting coil is aligned in parallel concerning the respective inclined misalignment angles of 15 degrees, 25 degrees, and 45 degrees of the receiver coil. Variation of mutual inductance concerning angular misalignment is shown in Table 4.

**Table 4:** Variation of mutual inductance concerning angular misalignment.

S. no.	Misalignment angle(in degrees)	Mutual inductance (in μH)
1	0	64.74
2	15	63.94
3	25	64.89
4	45	63.84

It also shows that the flux is distributed or focused along the middle of the coil. Aligning the transmitter coil according to the position of the receiver coil can help retain the efficiency of the system.

**Figure 14:**
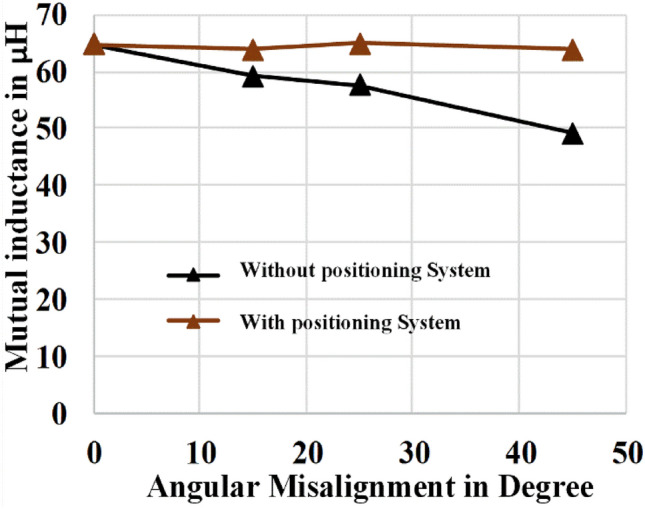
Comparison of mutual inductance.

The mutual inductance between the coil pair is compared with misalignment and without misalignment in the results obtained from the simulation, as shown in Fig. 14. The Value of mutual inductance between transmitter and receiver is around 65 ${\varvec{\mu}}{\varvec{H}}$ at zero misalignment. It gets decreases when angular misalignment occurs due to external facts. As illustrated in Fig. 14 the investigation suggests that the reduction of angular misalignment can maintain the mutual inductance at maximum value, which improves flux linkage, coupling coefficient, and capability of power transfer. The accurate estimation of mutual inductance is complex in practical case, therefore the investigation based on the simulation tool reduces the complexity of the investigation with minimum deviation from accurate value. Also the simulation results things to see the effect of angular misalignment in WPT system.

## Experimental verification with hardware setup

The investigations made with the theoretical as well as simulation is validated with prototype hardware implemented EV with wireless charging setup. The EV prototype is developed with integrating the designed parameters listed in Tables 1, 2. In WPT system decisive part is designing of the coils, due to high frequency operation the impact of skin effect and proximity effect is prominent which causes over heating of the coil wire also reduces the overall system efficiency. To defeat these issue, the transmitter and receiver coils are made with Litz wire. In general, Litz wire is made of insulated small twisted strands, the number of strands and its thickness depends on the frequency of operation. Based on the current and frequency requirement SWG of the Litz wire is selected, the coil shown in the prototype setup used 42 SWG sized Litz wire.

**Figure 15:**
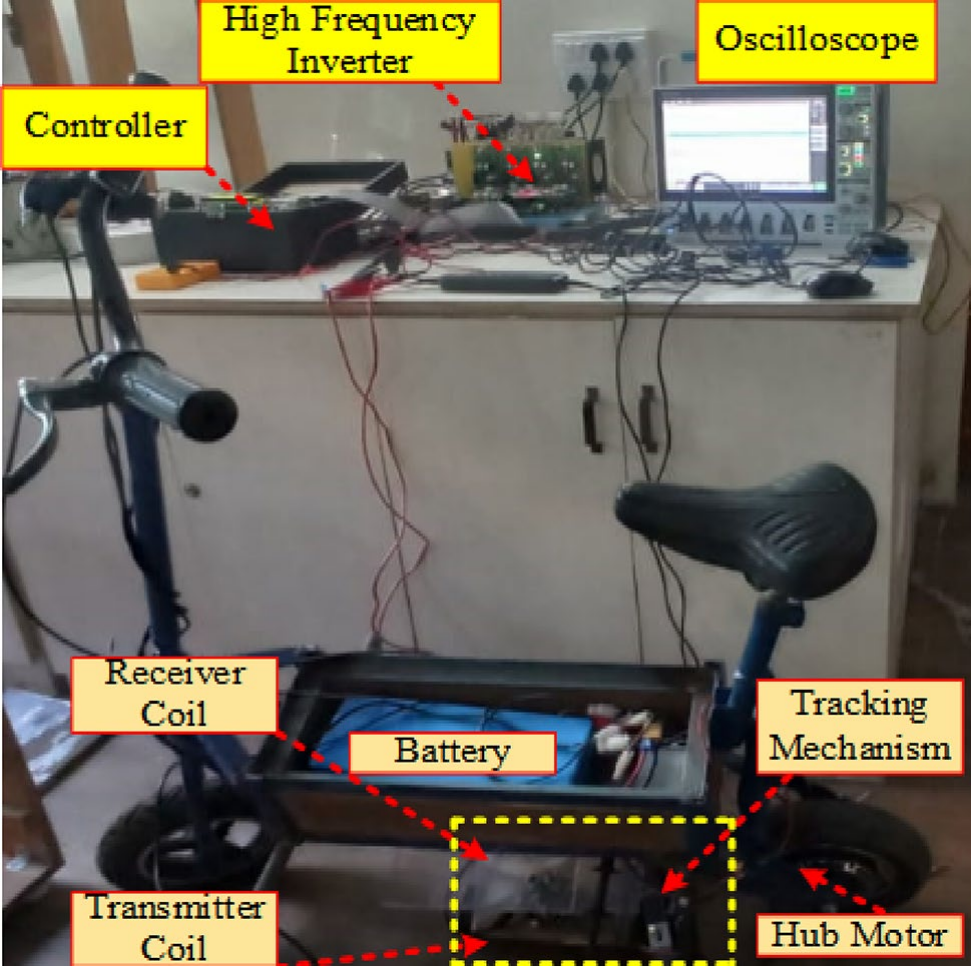
Proposed tracking mechanism.

Figure 15 portray the overall wireless charging system with designed prototype EV. The coupling pads are integrated with the proposed tracking mechanism. As per standard SAE-J2954 the WPT system is operated with 85 kHz. This high frequency AC is generated with 1 kW full bridge inverter made with semikron IGBT (SKM200GB125D) switch. This inverter is powered from grid supply of single phase 220 V AC with 50 Hz through a full bridge AC/DC diode based converter module (RITU 160) with filter capacitor (PG6TUPS). The switches were protected from high dv/dt by connecting snubber capacitor (KP3C). The required gate pulse for the high frequency inverter is generated by using SPATRON-6 FPGA controller. With series compensation the high frequency supply excites the primary coil which produces flux which further induces EMF in the secondary coil located at 160 mm vertical distance to the primary coil. The secondary induced EMF is resonated with secondary side compensation then which is converted into DC with AC/DC converter module. Finally, the regulated DC is connected to the Lithium based battery of capacity 24 V, 16 Ah.

Hard switching of inverter at resonant frequency obviously decreases the overall efficiency of the WPT system due to switching losses in the IGBTs. Therefore, soft switching either zero voltage switching (ZVS) or zero current switching (ZCS) is necessary. In this designed WPT system ZVS in incorporated in the inverter side. To achieve ZVS it is necessary to operate the inverter at a frequency which should slightly deviated from the resonant frequency. This deviated frequency operation of the inverter causes high conduction losses and further decreases the power transfer efficiency. Therefore, the appropriate control strategy is to be used which can maintain the both resonance condition in the transmitter and ZVS operation. Symmetrical clamped mode control strategy is used, which is also called as phase shift modulation control strategy. This control technique produces minimum switching losses and also maintains the resonant condition.

Generally, the circular type of pads is porn to the issues of misalignment, it is essential to eliminate the misalignment between transmitter and receiver pads. To aid with alignment, this system employs sensing coil and a servo motor. When the transmitter is appropriately positioned, its magnetic field generates an electrical current in the receiving coil. As a rectifier, the receiver's power management unit turns the current back into DC electricity, which is then utilized to charge the EV's battery. The energy received powers the vehicle's motor and auxiliary systems while also charging the battery, if one is available.

The proposal in Fig. 15 advocates for the use of ultrasonic sensors to explain the issue of angular misalignment in WPT systems, thereby improving the system's efficiency. Ultrasonic sensors employ sound waves to gauge the distance that exists between the receiver and the emitting coils. In the hardware setup two ultrasonic sensors were incorporated over the transmitter pads ensures that which covers entire dimensions of the circular pad. This sensor monitors the distance between the transmitter and receiver pad on either ends of the pads. To make the process easier IOT based devices such as Arduino is used to operate the drive mechanism to tilt the transmitter pad axially in accordance with receiver pad through a stepper motor. Finally, this proposed system resolves the angular misalignment issue and improves the power transfer efficiency of the wireless charging system.

In WPT based charging system high frequency source fed from inverter to the primary coil is pivotal. The waveform was supplied to the primary coil, which in turn produced an alternating magnetic field. This magnetic field induces an AC voltage in the secondary coil, facilitating the transfer of power wirelessly. The AC voltage generated by the secondary coil is subsequently rectified to charge the battery or power the intended device. To calculate the overall efficiency of the system, the input voltage, input current, output voltage, and output load are required. The overall efficiency of a power transfer system can be determined using the Eq. (54)

54 $$\eta = \frac{{{{V^{2} } \mathord{\left/ {\vphantom {{V^{2} } {R_{Load} }}} \right. \kern-0pt} {R_{Load} }}}}{{V_{in} \times I_{in} }}$$

**Figure 16:**
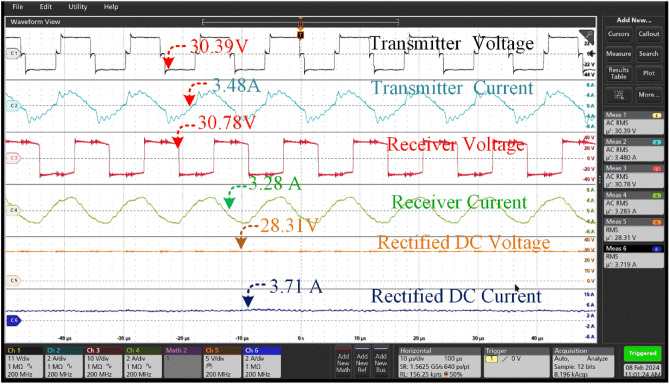
Transmitter voltage & current and Receiver voltage & current when at misalignment angle of 0° between the coil pair.

From Fig. 15, the receiver coil is aligned with a misalignment angle of 0 degrees concerning the transmitter coil. The provided scenario describes a wireless power transfer system that includes a transmitter coil, a receiver coil, and subsequent rectification for the purpose of charging a battery (Fig. 16).

The transmitter coil is producing a high-frequency alternating current (AC) in order to create a similar voltage in the receiver coil. The given figures of 30.39 V and 3.48A represent the voltage and current produced by the transmitter coil, respectively. This output is essential for commencing the wireless power transmission procedure.

The reception coil produces a marginally reduced voltage of 30.78 V and a little elevated current of 3.28A in comparison to the transmitter's output. The transmission efficiency, quantified at 94 percent, emphasizes the efficacy of wirelessly transferring electricity between the coils while minimizing any energy losses.

After being received by the receiver coil, the alternating current is converted into a direct current (DC) through rectification, which is then used to charge the battery. The rectifier unit maintains voltage within permissible parameters for charging reasons. The measured rectified voltage and current in this case are 28.31 V and 3.71A, respectively, suggesting a successful conversion process. The corrected output obtained from the receiver is subsequently employed to charge a battery possessing distinct characteristics. The battery possesses a capacity of 18Ah (ampere-hours) and a nominal voltage of 24 V.

The performance of the 00 misaligned charging system is determined by parameters like as efficiency, voltage regulation, and charging time. The transmission efficiency, denoted as 95 percent, is the degree of efficiency in transferring electricity from the transmitter to the receiver, accounting for unavoidable losses.

So, there is no need for the transmitter coil to align from its original angle. Thus, there is no power loss in the system and the efficiency remains the same.

**Figure 17:**
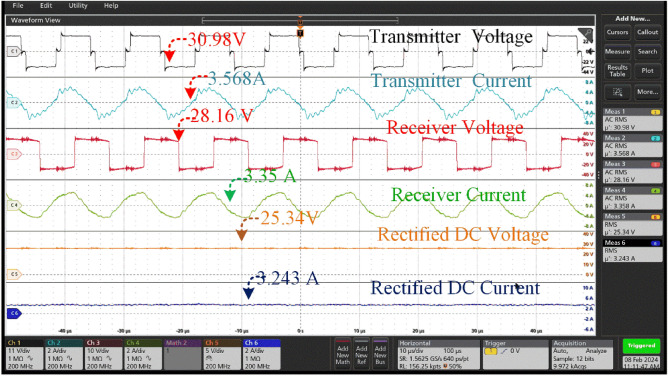
Transmitter voltage & current and Receiver voltage & current when at misalignment angle of 15° between the coil pair without tracking system.

The coil supplies a transmitter voltage of 30.98at a current of 3.56A. The transmission configuration guarantees optimal energy transfer to the receiver coil, which obtains high-frequency alternating current (AC) at a marginally lower voltage of 28.16 V and a slightly higher current of 3.35A. The transmission efficiency, which is a remarkable 85 percent, highlights the effectiveness of the energy transfer method used is shown in Fig. 17. Which is less compared 0^0^ of misalignment. Upon receiver input to the rectifier unit effortlessly turns the alternating current from the secondary coil into a rectified output for additional use. The rectification procedure results in a consistent output voltage of 25.34 V and a current of 3.243A, guaranteeing a dependable and unwavering power source for battery charging. The rectified voltage and current are precisely adjusted to charge the battery system that is capable of storing 18 Ah at a nominal voltage of 24 V. This charging configuration guarantees the best charging performance, efficiently restoring the battery's energy reserves for future usage in different applications, therefore promoting the smooth operation of the entire system.

**Figure 18:**
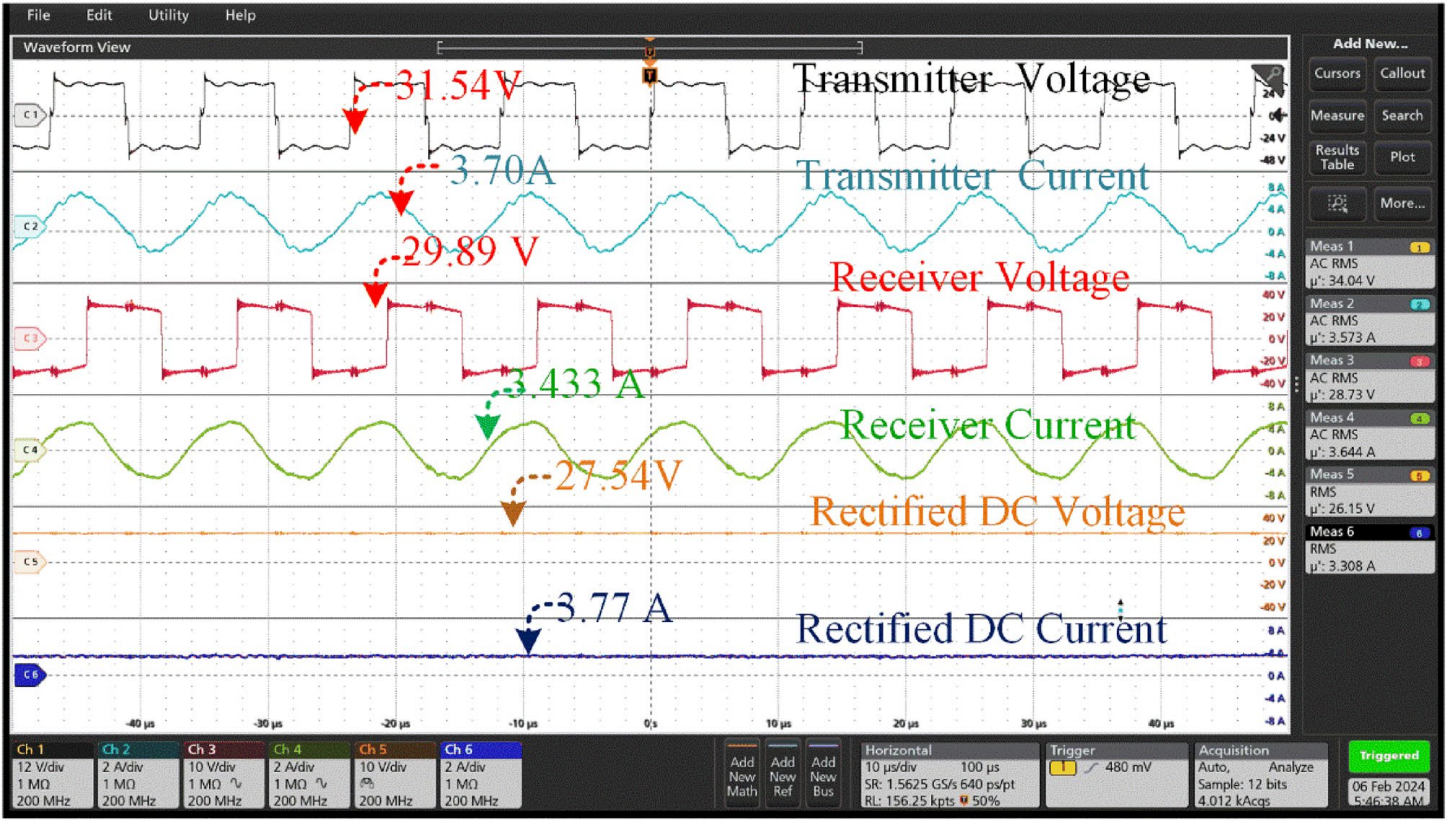
Transmitter voltage & current and Receiver voltage & current when at misalignment angle of 15° between the coil pair with tracking system.

The coil generates a voltage of 31.54 V and a current of 3.7A for the transmitter. The transmission configuration ensures efficient energy transfer to the receiver coil, resulting in the acquisition of high-frequency alternating current (AC) at a slightly reduced voltage of 29.89 V and a slightly increased current of 3.433A. The energy transfer mechanism used demonstrates its effectiveness through a notable transmission efficiency of 88 percent, as depicted in Fig. 18. The number of misalignments between the transmitter and reception coil is greater than 150. The rectification process yields a stable output voltage of 27.54 V and a current of 3.77A.

Figures 17, 18 provide a comparison between the system without tracking and the system with tracking. The power losses are diminished in comparison to the misaligned state. In this case, the power loss is little in comparison to a misalignment of 0 degrees. According to Fig. 16, the transmitter coil is positioned in parallel with the inclined angle of the receiver coil. This implies that the transmitter coil is aligned at a 15-degree angle from its original position in order to be parallel with the reception coil.

**Figure 19:**
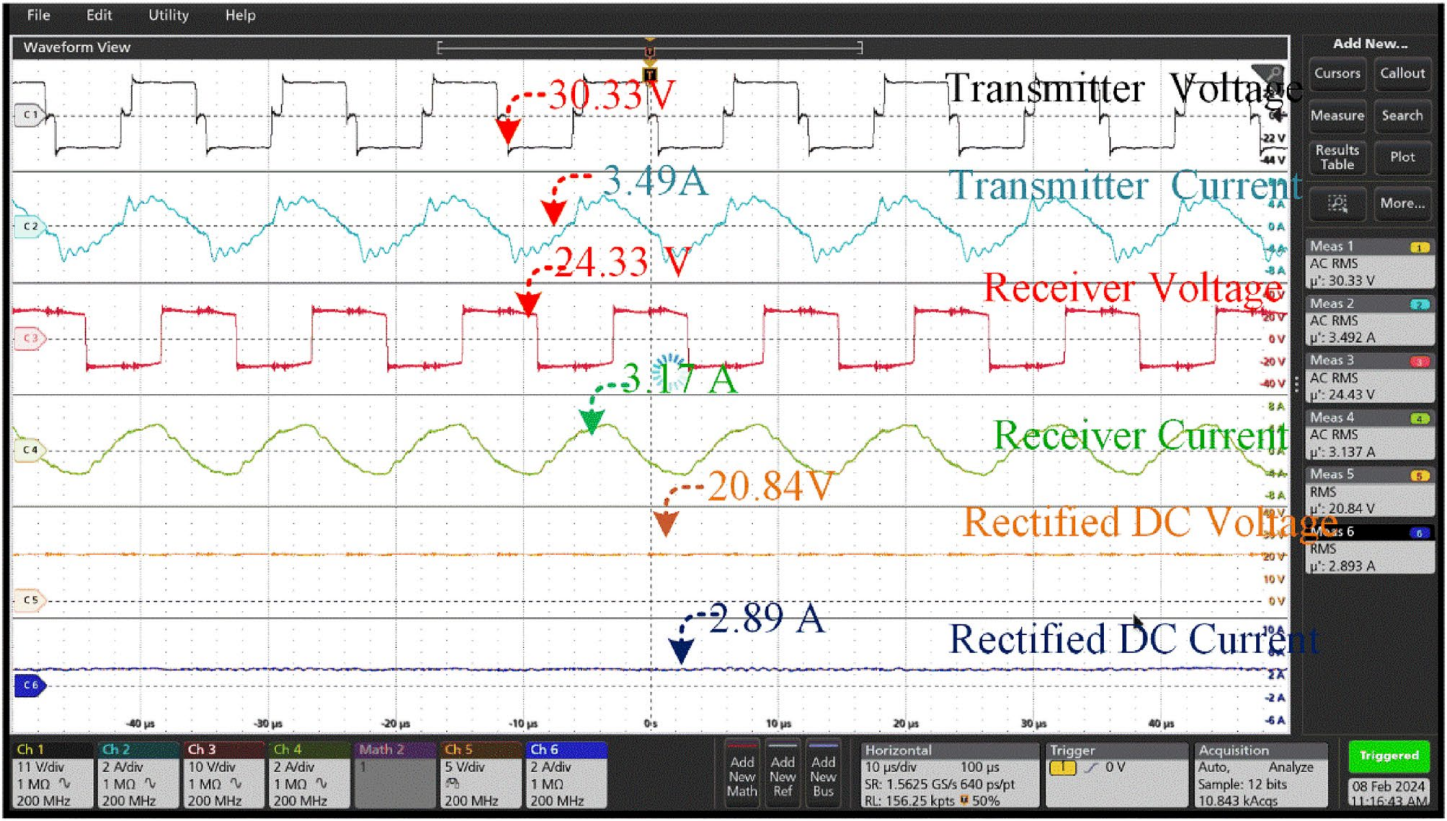
A Transmitter voltage & current and Receiver voltage & current when at misalignment angle of 25° between the coil pair without tracking system.

Under the 25^0^ of the misalignment t of 30.33 V at a current of 3.49A. The transmission configuration guarantees a lower voltage of 24.33 V and a slightly higher current of 3.17A is shown in Fig. 19. The rectification procedure results in a consistent output voltage of 20.84 V and a current of 2.89 A which is insufficient to charge the battery that is connected for charging. At a current of 3.75A, the aligned state yields a voltage of 31.43 V, which is within the range of 250. The transmission configuration ensures a voltage of 29.99 V, which is lower, and a current of 3.44A, which is slightly greater. The rectification process yields a stable output voltage of 27.67 V and a current of 3.78 A, which is utilized for charging the associated battery which is shown in Fig. 19.

The maximum misalignment of 45^0^ the charging port of the battery receives 16.21 V which is not sufficient for charging the good battery available at the charging. However, the unused battery is connected, and the charging current measured at 2.19A is shown in Fig. 21. At the misalignment of 45^0^ it is observed the not suitable for charging. The coil tracing mechanism at the 45^0^ offers a charging voltage of 26.99 V with a charging current of 3.73 V by tracking the transmitter coil, is shown in Fig. 22.

**Figure 20:**
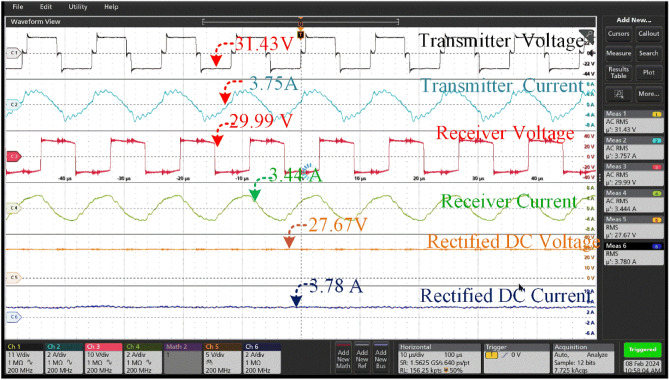
Transmitter voltage & current and Receiver voltage & current when at misalignment angle of 25° between the coil pair with tracking system.

**Figure 21:**
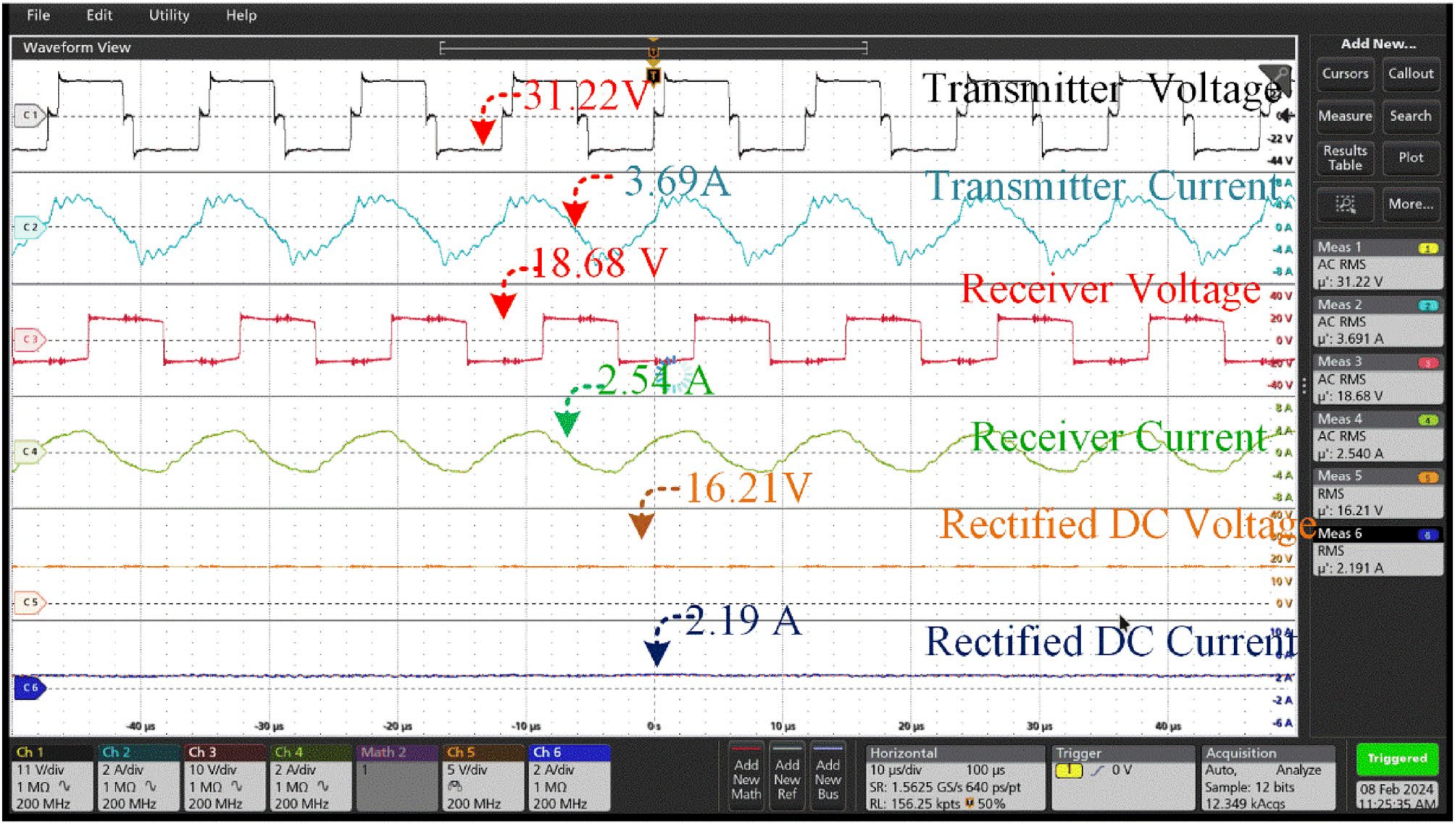
Transmitter voltage & current and Receiver voltage & current when at misalignment angle of 45° between the coil pair without tracking system.

**Figure 22:**
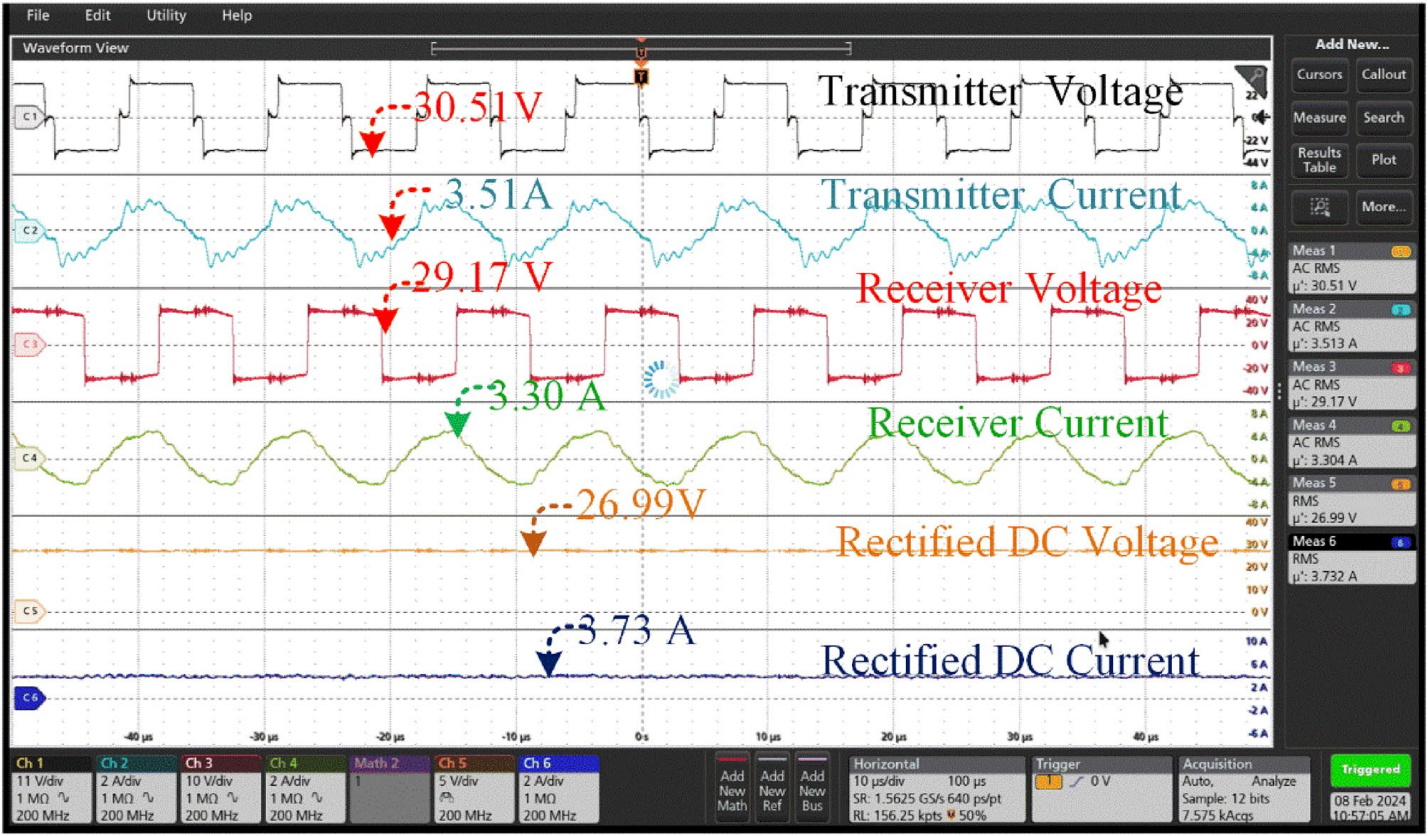
Transmitter voltage & current and Receiver voltage & current when at misalignment angle of 45° between the coil pair with tracking system.

Thus, the hardware results of the voltage and current across the Transmitter and Receiver coils at different angular misalignment conditions between the coil pair are shown in Figs. 16, 17, 18, 19, 20, 21, 22. This shortfall of power transmission is improved by aligning the receiver coil concerning the angle of misalignment. By aligning the transmitter coil according to the angle inclination of the receiver coil the power transmission is improved were shown in the figures. The increase in the angle between the coil pair significantly reduces the flux distribution in the center of the coil, which can be seen from the figures. The unequal distribution of flux in the receiver coil significantly reduces the mutual inductance between the coil pair. Thus, the power loss increases with the reduction of mutual inductance.

**Figure 23:**
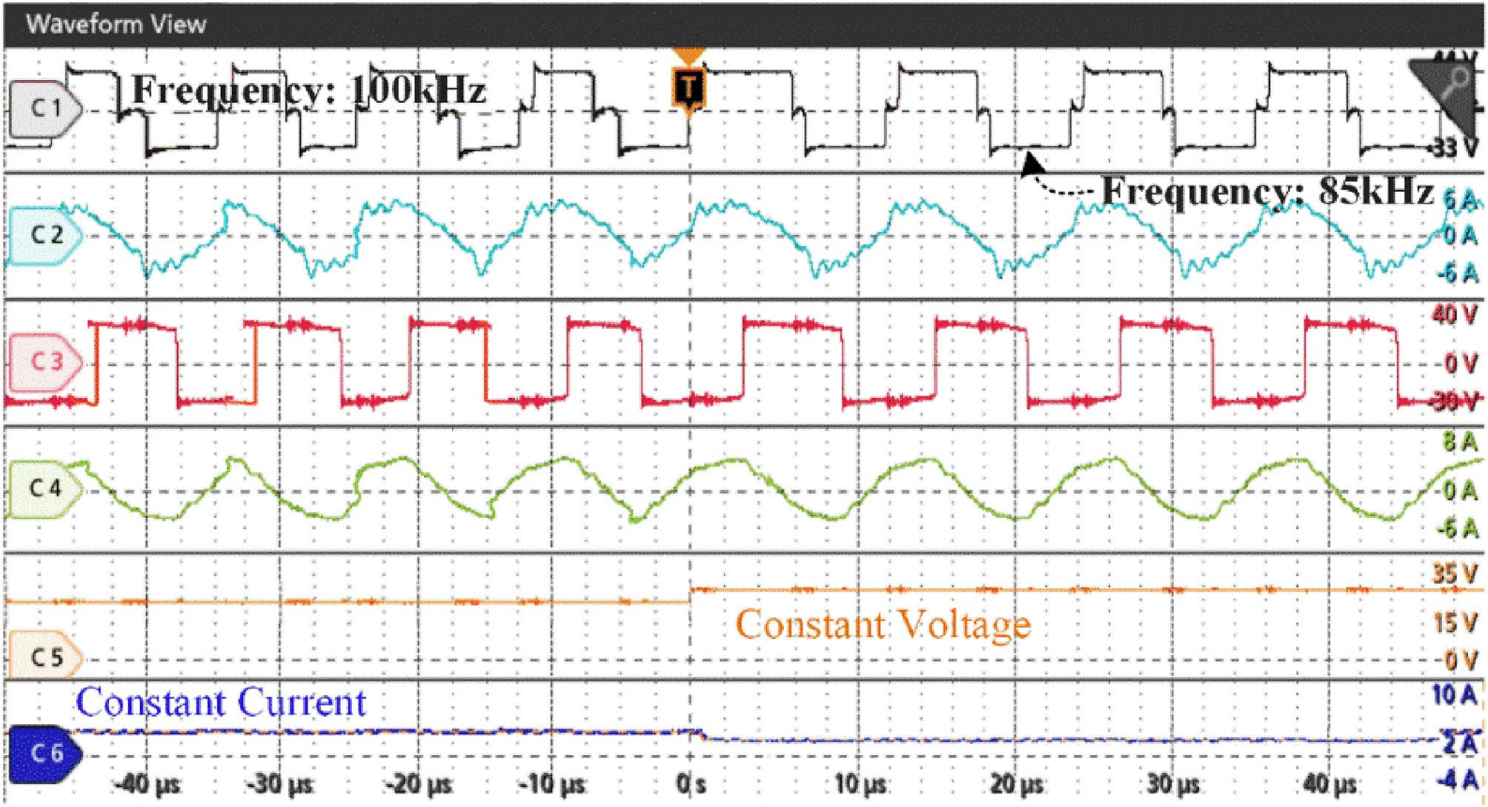
Experimental charging input from CC mode to CV mode.

Figure 23 illustrates the transition from constant current to constant voltage charging (f_CC_ to f_CV_), showing a change in the driving signal of the H-bridge inverter from 100 to 85 kHz. This modification induces a discernible albeit minor variation in both the voltage and current during the switching process. This transition is a crucial point in the charging process, signifying the change from emphasizing the supply of a constant electric current to maintaining a stable voltage output. This ensures that the system is charged efficiently and in a controlled manner.

As the angle of misalignment increases, there is a reduction in mutual inductance, which was shown through the simulation using ANSYS MAXWELL software. This chapter also summarizes the proof with the experimental verification that the reduction in efficiency, reduces due to the decrease in mutual inductance. It also proves that the power loss is also more with the increase in misalignment angle. Table 5 illustrates the output charging voltage and charging current at different cases of angular misalignment (15°, 25°, and 45°).

**Table 5:** Hardware output of positioning system.

Angular misalignment in degree	With positioning system		Without positioning system	
	Charging voltage in V	Charging current in A	Charging voltage in V	Charging current in A
0	28.31	3.71	NA	NA
15	27.54	3.77	25.34	3.24
25	27.67	3.78	20.84	2.89
45	26.99	3.73	16.21	2.19

It is observed from the table that the positioning system reduces the power loss due to angular misalignment. In the case of an electric scooter type of vehicle, there is the possibility of more than 40° misalignment due to its physical arrangement, in such case the power transfer efficiency may be reduced drastically.

**Figure 24:**
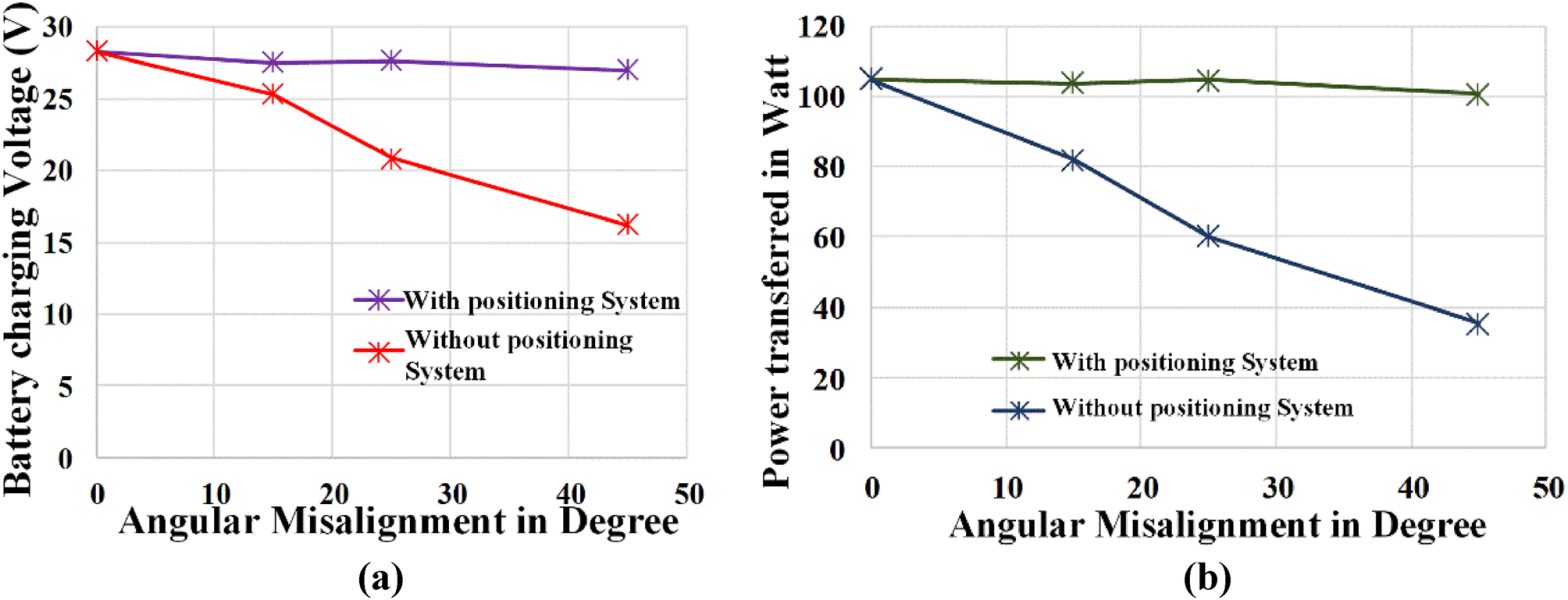
Influence of Pad Positioning system in (**a**) Charging Voltage (**b**) Power Transferred by WPT system.

From Fig. 24 it is observed that the voltage and power curve ensures that the power transfer capability of the WPT system is maintained at the same percentage even at 45° angular misalignment. With this proposed pad positioning system, the maximum flux distribution point is always maintained at the center of the circular pad.

### Loss analysis in the WPT system

In WPT system foremost losses were takes place in three parts: Inverter, Couplers, and Rectifier. The inverter losses involves switching losses (P_SL_) and conduction losses (P_CL_) which are expressed in Eqs. (55, 56), in the case of high frequency operation influence of P_SL_ is dominant ^35^

55 $$P_{SL} = 2V_{in} (i_{P} + i_{a} )\sqrt 2 \cos \left( {\frac{\theta }{2}} \right)f\left( {\frac{{e_{sw,on} + e_{sw,off} }}{{V_{R} I_{R} }}} \right)$$

56 $$P_{CL} = \frac{{r_{ds} }}{\pi }(i_{P}^{2} + i_{a}^{2} )(\pi + \theta + \sin \theta )$$

The WPT coupler primarily involves copper losses (P_cu_) and core losses in Ferrite (P_fe_). Because of the high frequency operation losses owing to skin effect and proximity effect adds considerable value in addition to I^2^R^36^. The losses owing to skin effects and proximity effects are given in the following expression (57, 58).

57 $$P_{se} = n_{st} R_{dc} F_{R} (f)\left( {\frac{{I_{peak} }}{{n_{st} }}} \right)^{2}$$

58 $$P_{pe} = n_{st} R_{dc} G_{R} (f)\left( {H_{e}^{2} + \frac{{I_{peak}^{2} }}{{2\pi^{2} d_{a}^{2} }}} \right)$$

In WPT ferrite core were used to improve the power transmission and also minimize the leakage flux. The losses associated with ferrite is maximum in bi-polar coils, due to hysteresis effect. In this designed WPT system unipolar coil has preferred, the expression for the losses associated due to ferrite core is expressed in the following Eq. (59).

59 $$P_{fe} = k_{s} f^{\alpha } B^{\beta }$$

In general the losses in the diode bridge rectifier is minimum owing to low forward voltage drop when compare to power switches used in the inverter. But at high frequency operation the losses corresponding to the rectifier is high when compare to soft switched inverter. Figure X illustrates the power losses involved in WPT system due to aforesaid three major components such as inverter, coupler, and rectifier.

**Figure 25:**
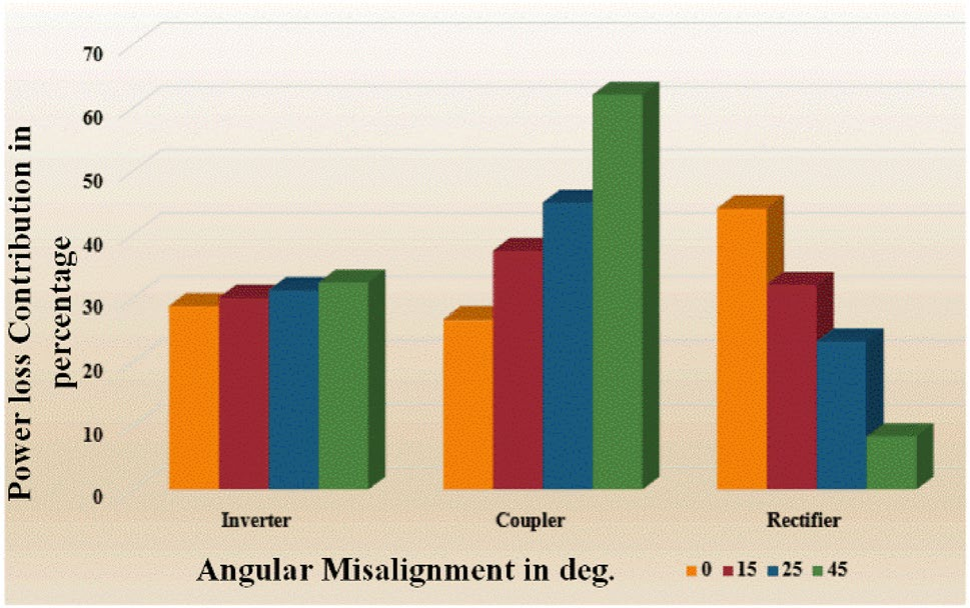
Power loss contribution with considered angular misalignment.

It is clear from the Fig. 25, when at zero misalignment the contribution of losses associated with rectifier is maximum owing to high frequency operation. When angular misalignment occurs the transferring power that gets decreased due to the increase of the leakage flux. The proposed tracking system which minimizes the leakage flux and enhances the power transfer efficiency.

**Figure 26:**
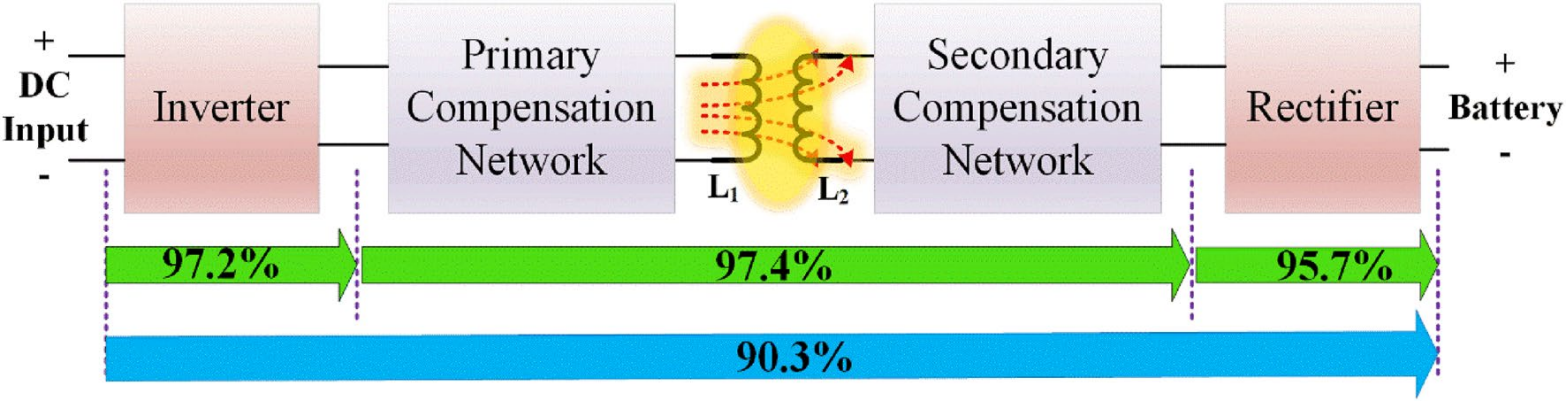
Efficiency of the proposed WPT system.

The proposed tracking system maintains the proper alignment between the transmitter and receiver coil. Figure 26 shows the efficiency distribution of the WPT EV charging system, inverter and coupler has better efficiency because of soft switching, compensation and the proposed tracking system. Moreover the tracking system uses single stepper motor of 3A, 12 V rating which consumes 36W of power only when the misalignment occurs. Therefore, the proposed tracking system enhances the angular misalignment with minimal power and cost.

Table 6 likely presents various research articles that focus on misalignment issues. However, despite the presence of several articles, none of them were addressed angular misalignment specifically. Despite the lack of research on angular misalignment in the existing literature, this article does tackle this issue. This article presumable introduces a method to mitigate the effects of angular misalignment. This article addressing angular misalignment achieves an efficiency of 90.3% in DC-DC conversion. This efficiency figure indicates how effectively the system converts input DC power to output DC power and charges the EV battery, especially if angular misalignment was a significant challenge that needed to be overcome.

**Table 6:** Comparison with existing methodologies.

References	Efficiency	Method	Enhancement of angular misalignment
^37^	97.56	Novel mechanical structure	✗
^38^	89.1	Using passive components	✗
^39^	90%	Sensing primary current	✗
^40^	90.1	Using impedance and resonant frequency	✗
^41^	91.23	Intermittent coil	✗
**This article**	90.3%	Sensing coil	✓

## Conclusion

A wireless charging system employing inductive technology for electric vehicles has been developed to examine the impact of angular misalignment between coils. The Wireless Power Transfer technology analyzed changes in mutual inductance caused by angular misalignment using theoretical analysis. The Ansys-Maxwell FEA analysis software was used to generate a coil model with different axial offset angles using finite element analysis. The software computed the magnitude of the magnetic field generated by the coupling mechanism at the center location. When maintaining a consistent transmission angle, the magnetic shield demonstrated an effective capability to reduce the magnetic field intensity externally while simultaneously amplifying it within the coupling mechanism. A 3D test platform was built to verify the accuracy of theoretical analysis results and the effectiveness of the system simulation method in addressing angular misalignment problems in electric vehicles, namely in the Wireless Power Transfer system. Additionally, an electric scooter was developed to assess practical feasibility and evaluate the tracking mechanism. The tracking mechanism, operating at a consistent transmission distance, demonstrates its effectiveness in modulating the coil coupling mechanism to decrease and increase the magnetic field. The findings of this study offer valuable insights for guiding the design of future inductive wireless charging systems intended for Wireless Power Transfer in EV. Specifically, it suggests the implementation of a suitable tracking mechanism to mitigate angular misalignment issues.

## Data Availability

The datasets used and/or analysed during the current study are available from the corresponding author upon reasonable request.
